# Hepatic glutathione depletion ameliorates MASLD through selective protein oxidation and inhibition of lipogenesis

**DOI:** 10.1172/JCI197556

**Published:** 2026-04-15

**Authors:** Xiang-Yu Liu, Guoxiao Wang, Yingying Yu, Haopeng Xiao, Kentaro Oh-hashi, Xu Shi, Shuning Zheng, Robert Gerszten, C. Ronald Kahn

**Affiliations:** 1 Section of Integrative Physiology and Metabolism, Joslin Diabetes Center, and Department of Medicine, Harvard Medical School, Boston, Massachusetts, USA.; 2 Broad Institute of MIT and Harvard, Cambridge, Massachusetts, USA.; 3 Department of Biochemistry, and; 4 Stanford Cancer Institute, Stanford University School of Medicine, Stanford, California, USA.; 5 Department of Cancer Biology, Dana-Farber Cancer Institute, Boston, Massachusetts, USA.; 6 Department of Cell Biology, Harvard Medical School, Boston, Massachusetts, USA.; 7 Center for One Medicine Innovative Translational Research (COMIT), Institute for Advanced Study, and; 8 Department of Chemistry and Biomolecular Science, Faculty of Engineering, Gifu University, Yanagido, Gifu, Japan.; 9 Division of Cardiovascular Medicine, Beth Israel Deaconess Medical Center, Harvard Medical School, Boston, Massachusetts, USA.

**Keywords:** Cell biology, Hepatology, Metabolism, Metabolomics

## Abstract

Glutathione (GSH) maintains a reduced cellular environment and is widely believed to mitigate disease-associated oxidative damage to proteins, thereby protecting against metabolic dysfunction–associated steatotic liver disease (MASLD). However, this widely accepted assumption remains largely untested because of challenges in physiologically manipulating hepatic GSH levels during disease development. Here, we have utilized liver-specific overexpression of cation transport regulator homolog 1 (*Chac1*), a recently identified intracellular GSH-degrading enzyme, to induce hepatic GSH depletion during MASLD progression. Contrary to canonical doctrine, GSH depletion unexpectedly protects against MASLD by substantially decreasing hepatic lipogenesis and fibrosis without triggering an oxidative stress response. Mechanistically, GSH depletion does not cause global protein oxidation but instead selectively oxidizes and destabilizes fatty acid synthase while decreasing lipogenic gene expression at the transcriptional level, collectively suppressing lipogenesis. Interestingly, *Chac1* expression is decreased in livers of patients with MASLD, highlighting its potential therapeutic relevance. These findings revise the conventional view of GSH in protein redox and demonstrate that targeted redox manipulation through GSH depletion protects against MASLD.

## Abstract 2

Depleting glutathione in the liver surprisingly prevents fatty liver disease by selectively oxidizing certain proteins involved in fat production, rather than causing broad damage.

## Graphical abstract

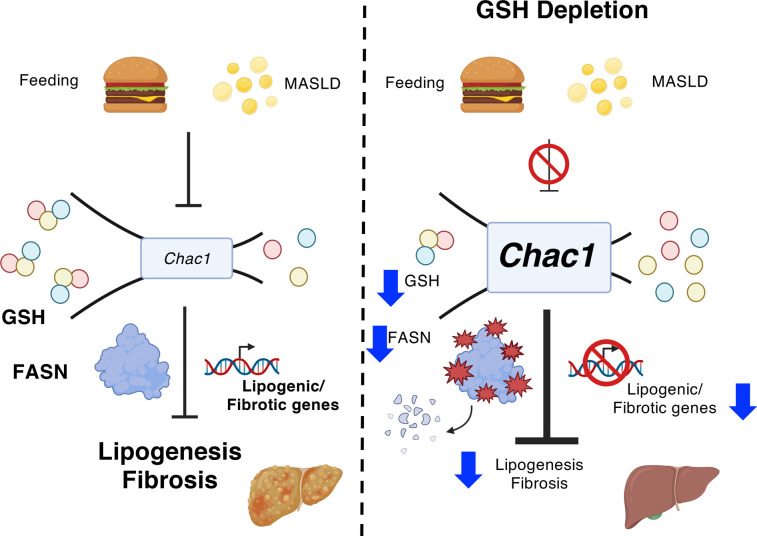

## Introduction

**Figure 1:**
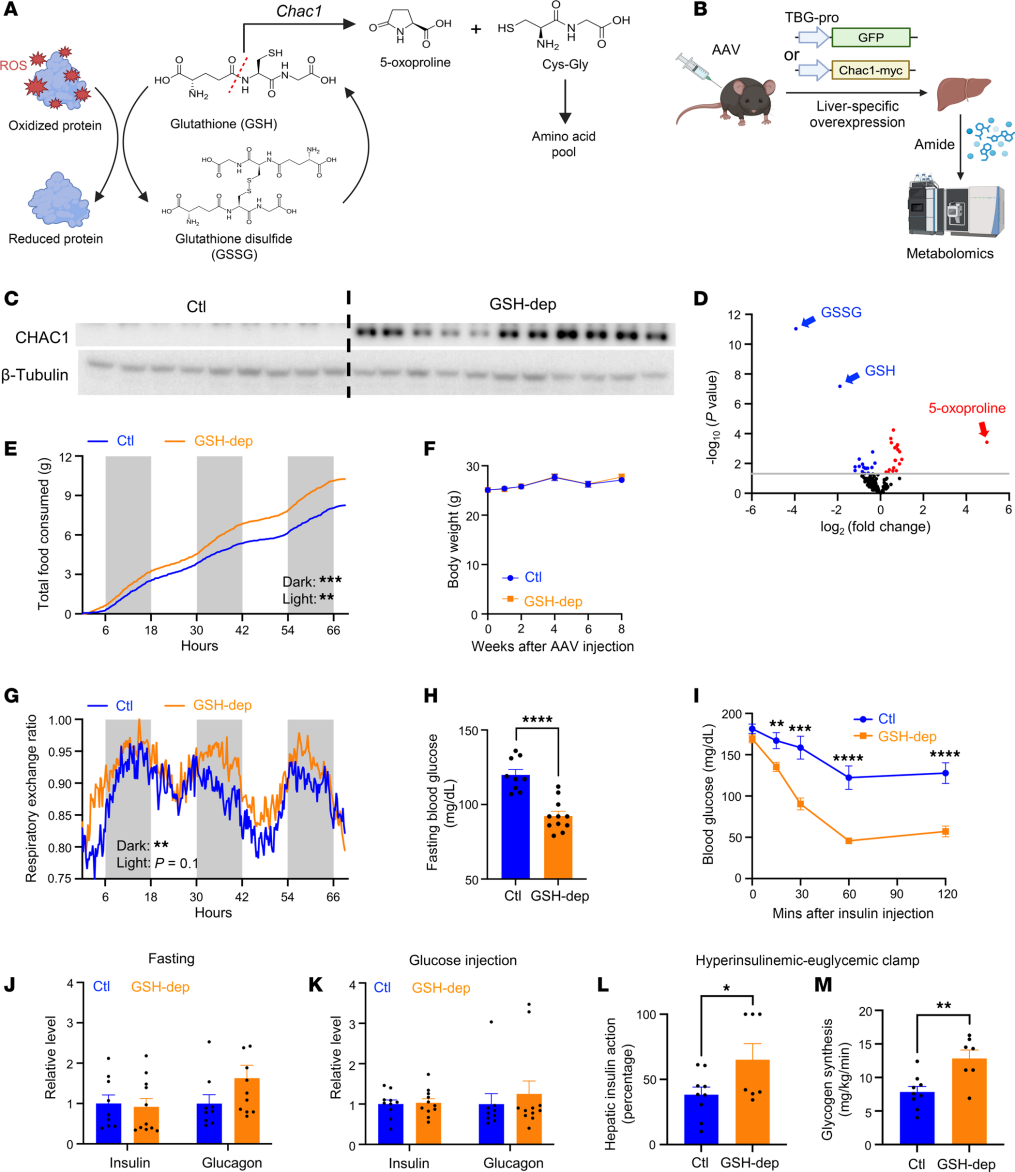
Hepatic GSH depletion improves insulin sensitivity. (**A**) The role of GSH and its depletion by *Chac1*. Cys-Gly, cysteinylglycine; ROS, reactive oxygen species. (**B**) Liver-specific *Chac1* overexpression using AAV8. (**C**) Overexpression of *Chac1* in liver assessed by immunoblot. (**D**) Metabolomic analysis on livers from control and hepatic GSH-depleted mice (*n* = 7). (**E**) Cumulative food intake by control or GSH-depleted mice (*n* = 6). (**F**) Body weights of mice after AAV injection (*n* = 9–11). (**G**) Respiratory exchange ratio of mice measured by metabolic cage (*n* = 6). (**H** and **I**) Blood glucose levels of mice after overnight fasting (**H**, *n* = 9–11) or during an insulin tolerance test (**I**, *n* = 8–10). (**J** and **K**) Relative levels of serum insulin and glucagon of mice during fasting (**J**) or after glucose injection (**K**) assessed using ELISA (insulin ELISA: Crystal Chem, 90080; glucagon ELISA: Crystal Chem, 81518). (**L** and **M**) Percentage of hepatic insulin action (**L**) and whole-body glycogen synthesis rate (**M**) of mice during hyperinsulinemic-euglycemic clamps (*n* = 7–9). *P < 0.05, **P < 0.01, ***P < 0.001, ****P < 0.0001. GSH-dep, GSH depletion.

Glutathione (GSH) is a tripeptide composed of cysteine, glycine, and glutamic acid. Within cells, GSH exists in 2 interconvertible states: the reduced form (GSH) and the oxidized form (GSSG). The conversion of GSH to GSSG is traditionally viewed as being coupled to the clearance of reactive oxygen species (ROS) and the reduction of oxidized proteins (Figure 1A), positioning GSH as a key antioxidant that globally mitigates oxidative stress on proteins (1–3). However, the physiological and pathophysiological roles of GSH remain largely unexplored in vivo, substantially limiting our ability to therapeutically target GSH in disease contexts.

GSH is primarily synthesized in liver, and decreased levels of circulating GSH have been reported in liver diseases, including metabolic dysfunction–associated steatotic liver disease (MASLD) and metabolic dysfunction–associated steatohepatitis (MASH) (4, 5). Together with the evidence of elevated oxidative stress in MASLD/MASH (6, 7), it has been widely assumed that hepatic GSH depletion is detrimental, leading to disease progression. Although this widely accepted assumption appears intuitive, it remains largely untested for several reasons. First, many studies have focused on plasma GSH levels or its constituent amino acids, which may not accurately reflect hepatic GSH status (4). Second, the reported decrease in plasma GSH levels among patients with MASLD has not been consistently reproduced across independent cohorts (4, 8). Third, and most importantly, the relationship between decreased GSH and MASLD is primarily based on association rather than demonstrated causality. Thus, the widely assumed detrimental effect of GSH depletion in MASLD remains largely unproven and lacks direct causal evidence.

Given the assumption that GSH depletion contributes to MASLD progression, GSH supplementation has been proposed as a therapeutic strategy. Clinical randomized controlled trials have begun to investigate the effects of GSH supplementation on MASLD/MASH. GSH has been administered through various routes, including intravenous and oral delivery. However, evidence supporting an increase in hepatic GSH levels following supplementation remains controversial (9), and the therapeutic outcomes for MASLD are inconclusive because of inconsistencies in study protocols and variable results across independent cohorts (10). Moreover, recent well-controlled animal studies have demonstrated that hepatic GSH may actually promote lipid accumulation and exacerbate MASLD (11–14), though the underlying molecular mechanisms remain largely undefined. Taken together, these findings indicate a need to critically reassess the role of GSH in hepatocyte function and development of MASLD.

GSH degradation has been believed to occur primarily in the extracellular space. After being exported from cells by multidrug resistance protein, extracellular and circulating GSH is hydrolyzed by γ-glutamyltransferase (GGT), an enzyme localized at the outer surface of cell membranes, to produce glutamic acid and cysteinylglycine (Cys-Gly) (15, 16). More recently, an intracellular GSH degradation pathway has been identified, catalyzed by the enzyme cation transport regulator homolog 1 (*Chac1*). *Chac1* is highly conserved among species (Supplemental Figure 1A; supplemental material available online with this article; https://doi.org/10.1172/JCI197556DS1) and specifically degrades GSH to 5-oxoproline and Cys-Gly (17–19). A paralog of *Chac1* in the mammalian genome is *Chac2*. Although *Chac2* has also been proposed to function as a GSH-degrading enzyme based on its approximately 50% sequence similarity to *Chac1*, it shows about 20-fold lower catalytic efficiency than *Chac1* on GSH degradation (20). Consequently, *Chac2* is mostly considered to serve as an enzyme for slow turnover of intracellular GSH or potentially to have other primary enzymatic functions. Furthermore, in liver, *Chac1* protein is moderately expressed, whereas *Chac2* protein is undetectable (21). Thus, *Chac1* is likely to function as the major enzyme mediating intracellular GSH degradation in liver.

In this study, to examine the role of hepatic GSH depletion on protein oxidation and MASLD progression, we have overexpressed *Chac1* specifically in liver to induce GSH depletion. Surprisingly, our data reveal that GSH depletion protects against MASLD progression, and it does not induce general oxidative stress response. Mechanistically, in contrast with the canonical doctrine that GSH globally maintains proteins in a reduced state, we show that GSH depletion selectively oxidizes and promotes the degradation of fatty acid synthase (FASN), a key regulator of lipid metabolism. In addition, GSH depletion also suppresses lipogenesis by downregulating key lipogenic genes at the transcriptional level, further protecting against MASLD.

## Results

### Hepatic GSH depletion is achieved through Chac1 overexpression.

*Chac1* functions as a GSH-specific γ-glutamylcyclotransferase, catalyzing the degradation of GSH into 5-oxoproline and Cys-Gly, thereby decreasing intracellular GSH levels (Figure 1A) (17, 19). To achieve GSH depletion in liver, we engineered an adeno-associated virus (AAV) vector encoding *Chac1* fused to a Myc tag, driven by the thyroxine-binding globulin (TBG) promoter to achieve liver-specific overexpression of *Chac1* (Figure 1B). AAV vectors expressing GFP served as negative controls. qRT-PCR and immunoblot analyses confirmed robust overexpression of *Chac1* in liver (Supplemental Figure 1B and Figure 1C). Metabolomic profiling of liver extracts revealed significant and specific decreases in both reduced (GSH) and oxidized (GSSG) glutathione levels, accompanied by an increase of 5-oxoproline, indicating effective GSH degradation by functional *Chac1* (Figure 1D and Supplemental Figure 1C). Circulating GSH levels were also significantly decreased (Supplemental Figure 1D), indicating liver is the primary organ supplying GSH to the circulation. Interestingly, markers of oxidative stress response, including *Nrf2* and *Keap1*, remained unchanged in liver (Supplemental Figure 1E). Consistent with this, *Chac1* overexpression in hepatocytes did not change the ROS levels (Supplemental Figure 1F). Thus, these data indicate that *Chac1*-mediated GSH depletion does not elicit a compensatory oxidative stress response.

### Hepatic GSH depletion increases insulin sensitivity.

Mice with hepatic GSH depletion exhibited increased food intake compared with controls (Figure 1E), while body weight, physical activity, and energy expenditure remained unchanged (Figure 1F and Supplemental Figure 1, G and H). Tissue weights of liver, adipose tissue, and skeletal muscle were also comparable between groups (Supplemental Figure 1, I–N). Serum alanine aminotransferase (ALT) levels were similar, indicating GSH depletion did not cause overt liver injury (Supplemental Figure 1O). Interestingly, GSH-depleted mice displayed a significantly elevated respiratory exchange ratio (RER) (Figure 1G), indicating increased carbohydrate utilization. Consistent with this metabolic shift, these mice exhibited lower blood glucose levels both during fasting and insulin tolerance testing (Figure 1, H and I). The levels of insulin and glucagon during fasting or after glucose injection were comparable between GSH-depleted and control mice (Figure 1, J and K), indicating that the observed differences in blood glucose were not due to altered levels of the main glucose-controlling hormones. We therefore hypothesized that the decreased blood glucose levels in GSH-depleted mice resulted from increased insulin sensitivity. To test this directly, we performed hyperinsulinemic-euglycemic clamp studies (Supplemental Figure 1P). Indeed, GSH-depleted mice demonstrated a higher level of hepatic insulin action (Figure 1L), and increased whole-body glycogen synthesis (Figure 1M), supporting enhanced insulin sensitivity as a consequence of GSH depletion. Together, these data indicate that hepatic GSH depletion increases insulin sensitivity with enhanced carbohydrate utilization.

### Hepatic GSH depletion protects against high-fat diet–induced MASLD.

**Figure 2:**
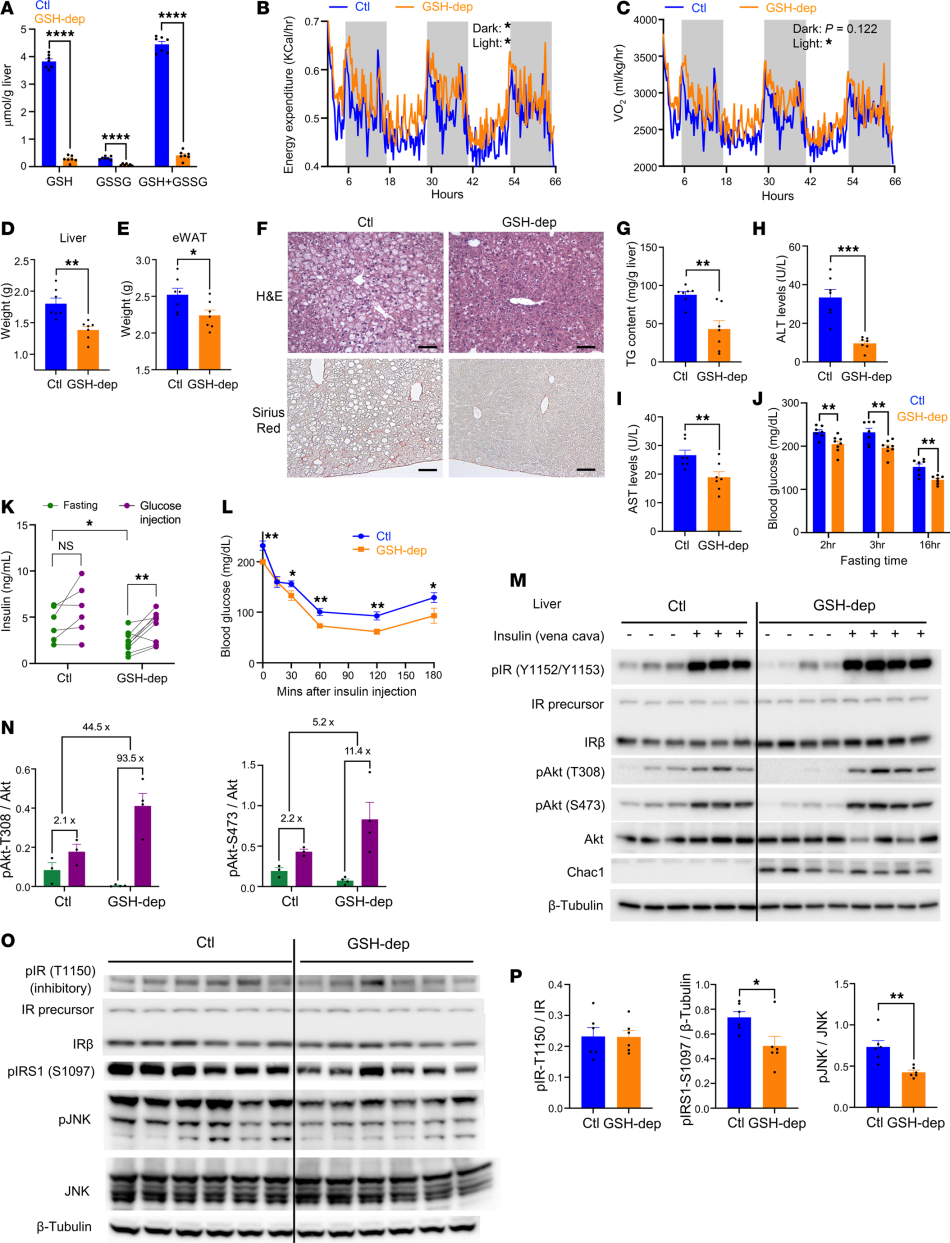
Hepatic GSH depletion protects against HFD-induced MASLD. (**A**) Levels of GSH and GSSG in livers of mice measured using Cayman Chemical Glutathione Assay Kit. (**B** and **C**) Energy expenditure (**B**) and oxygen consumption rate (**C**) of mice (*n* = 5). (**D** and **E**) Weights of liver (**D**) and eWAT (**E**) in control or GSH-depleted mice. (**F**) H&E and Sirius red staining in the liver. Scale bar: 80 μm. (**G**) Quantitative analysis of triglyceride (TG) levels in the liver. (**H** and **I**) Circulating levels of ALT (**H**) and AST (**I**) in mice. (**J**) Blood glucose levels of mice during fasting. (**K**) Levels of serum insulin during fasting or after glucose injection. (**L**) Blood glucose levels of mice during insulin tolerance test (*n* = 7). (**M**) Protein levels of key downstream effectors of insulin signaling in livers after insulin injection through vena cava. (**N**) Quantitative analysis of immunoblots in **M**. (**O**) Protein levels of inhibitory regulators of insulin signaling in livers. (**P**) Quantitative analysis of immunoblots in **O**. Mice were fed an HFD for 2 weeks before infection with AAV and were sacrificed 8 weeks later. *P < 0.05, **P < 0.01, ***P < 0.001, ****P < 0.0001. p, phosphorylated.

To investigate the effect of GSH depletion on the development of MASLD, mice were subjected to high-fat diet (HFD) feeding containing 60% fat. As in chow-fed mice, hepatic GSH levels were significantly decreased by approximately 90% following *Chac1* overexpression (Figure 2A). Compared with control mice, GSH-depleted mice exhibited similar body weight gain (Supplemental Figure 2A) and food intake (Supplemental Figure 2B), a mild increase of carbon dioxide production (Supplemental Figure 2C), and similar RER (Supplemental Figure 2D) and locomotor activity (Supplemental Figure 2E). However, mice with depletion of hepatic glutathione showed significantly elevated whole-body energy expenditure (Figure 2B) and oxygen consumption (Figure 2C), indicating increased metabolic rate. Consistent with these findings, GSH-depleted mice showed significantly decreased weights of liver and epididymal (visceral) white adipose tissue (eWAT) (Figure 2, D and E), while weights of inguinal (subcutaneous) white adipose tissue (iWAT), brown adipose tissue (BAT), or skeletal muscle remained unchanged (Supplemental Figure 2, F–H). Histological and biochemical analysis of liver revealed marked decreases in hepatic lipid accumulation, lipid droplet formation, and fibrosis in GSH-depleted mice (Figure 2, F and G). These were accompanied by lower serum levels of ALT and aspartate aminotransferase (AST) (Figure 2, H and I), indicating protection against MASLD-induced liver injury. Similarly, eWAT from GSH-depleted mice displayed substantially decreased fibrosis and smaller adipocyte size, indicating decreased lipid storage and MASLD-associated inflammation in eWAT (Supplemental Figure 2I). GSH-depleted and control mice showed similar levels of circulating free fatty acid, triglyceride, and glycerol (Supplemental Figure 2, J–L), indicating that the decreased lipid storage in liver and eWAT was not accompanied by hyperlipidemia.

In addition to inducing fatty liver, HFD also promotes systemic metabolic syndrome with insulin resistance and glucose intolerance. Under HFD condition, GSH-depleted mice exhibited significantly lower fasting blood glucose levels (Figure 2J), decreased fasting insulin levels, and improved glucose-stimulated insulin secretion (Figure 2K), all indicative of enhanced insulin sensitivity. This was further supported by insulin tolerance tests, which demonstrated significantly improved glucose lowering in GSH-depleted mice (Figure 2L). To directly assess insulin signaling, mice were administered insulin via the vena cava, followed by immunoblotting of key downstream effectors in liver and eWAT. In livers of GSH-depleted mice, the levels of pAKT at T308 and S473 were lower under basal conditions but showed a greater fold increase upon insulin stimulation compared with control mice under HFD (Figure 2, M and N). A similar pattern was observed in eWAT, where pAKT at T308 displayed a lower basal level but a greater insulin-induced response (Supplemental Figure 2, M and N).

Excessive hepatic lipid accumulation has long been proposed to impair insulin signaling pathway and promote insulin resistance. Thus, we hypothesized that GSH depletion enhances insulin sensitivity by decreasing lipid-induced inhibitory events on insulin signaling. While lipid accumulation has multiple effects on cellular signaling, 3 major lipid-associated inhibitory phosphorylation events on the insulin signaling pathway include (a) acylglycerol-induced pIR at T1150 (human T1160) (22), (b) fatty acid–induced pIRS1 at S1097 (human S1101) (23), and (c) ER stress–mediated pJNK (24). We then examined these 3 events in GSH-depleted liver by immunoblotting. In HFD-fed mice, GSH depletion did not significantly decrease pIR T1150, but it significantly decreased levels of pIRS1 at S1097 and pJNK (Figure 2, O and P), indicating that attenuation of these 2 inhibitory nodes may contribute to the improved insulin sensitivity.

Taken together, these findings indicate that hepatic GSH depletion confers substantial protection against the development and progression of HFD-induced MASLD/MASH, with improved insulin sensitivity.

### Hepatic GSH depletion suppresses lipogenesis and fibrosis through hepatocyte-intrinsic mechanisms.

**Figure 3:**
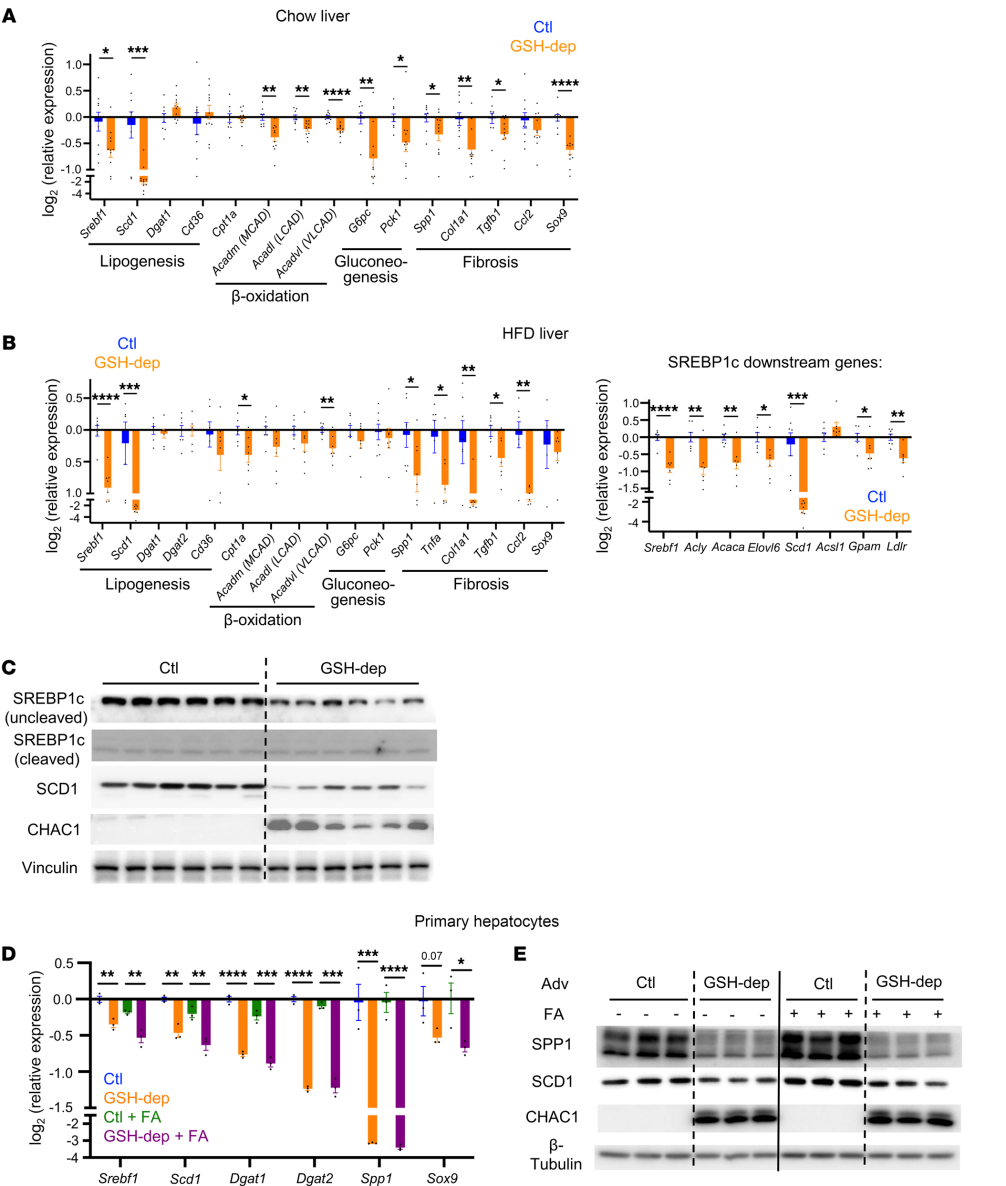
Hepatic GSH depletion suppresses lipogenesis and fibrosis. (**A**) Relative expression levels of key genes controlling lipogenesis, β-oxidation, gluconeogenesis, and fibrosis in livers of mice under chow diet. Expression was assessed by qRT-PCR (*n* = 9–11). Mice were fed a chow diet and were sacrificed 8 weeks after infection with AAV. (**B**) Relative expression levels of key genes controlling lipogenesis, β-oxidation, gluconeogenesis, and fibrosis in livers of mice after 10 weeks on HFD. Expression was assessed by qRT-PCR (*n* = 7). Mice were fed an HFD for 2 weeks before infection with AAV and were sacrificed 8 weeks later. (**C**) Protein levels of SREBP-1c, SCD1, and *CHAC1* in livers of mice under HFD. (**D**) Relative expression levels of *Chac1*-regulated genes in primary hepatocytes under 100 μM free fatty acid (FA) treatment. Expression was assessed by qRT-PCR (*n* = 3). Adenovirus (Adv): 400 μL/mL medium. Cells were harvested 3 days postinfection. (**E**) Protein levels of SPP1, SCD1, and CHAC1 in primary hepatocytes under 100 μM free FA treatment. Primary hepatocytes were infected with Adv overexpressing GFP or *Chac1*. Adv: 400 μL/mL medium. Cells were harvested 3 days postinfection. *P < 0.05, **P < 0.01, ***P < 0.001, ****P < 0.0001.

To identify the transcriptional adaptations underlying the metabolic changes induced by GSH depletion, we performed qRT-PCR to quantify the expression of key regulators of lipid and glucose metabolism, as well as fibrosis, in liver tissues from mice fed either a chow diet or HFD. The transcription factor (TF) *Srebf1* (SREBP-1c) plays a central role in lipogenesis, and it is activated by insulin, glucose, or fatty acids, with its expression level maintained by itself as an autoregulatory feed-forward loop during lipogenesis (25, 26). GSH depletion significantly decreased the hepatic expression of *Srebf1* and its direct downstream effector stearoyl-CoA desaturase (*Scd1*) under both chow and HFD conditions (Figure 3, A–C), indicating suppressed lipogenesis. In addition, under HFD conditions, most SREBP-1c downstream genes contributing to hepatic lipid accumulation were downregulated (Figure 3B). To determine whether decreased hepatic lipid accumulation could also be attributed to enhanced fatty acid β-oxidation, we examined the expression of key enzymes involved in this pathway, including *Cpt1a*, *Acadm* (MCAD), *Acadl* (LCAD), and *Acadvl* (VLCAD). However, GSH depletion led to either unchanged or decreased mRNA levels of these enzymes under both diets (Figure 3, A and B), indicating that fatty acid utilization was not upregulated. Thus, these data indicate that decreased lipid accumulation by GSH depletion is primarily due to diminished lipogenesis rather than increased fatty acid utilization.

Consistent with decreased fasting glucose levels and improved insulin tolerance observed under chow, and to a lesser extent under HFD conditions (Figure 1, H and I, and Figure 2, J and L), GSH depletion significantly downregulated gluconeogenic enzymes G6pc and Pck1 in chow-fed mice, with no substantial change under HFD (Figure 3, A and B).

Liver fibrosis is a key marker of MASLD/MASH progression, initiated by elevated hepatocyte expression of pro-fibrotic TFs such as *Sox9* and secreted factors such as *Spp1* (osteopontin) and *Tgfb1* (TGF-β1). These factors activate hepatic stellate cells and fibroblasts to produce extracellular matrix proteins involved in collagen synthesis, with *Col1a1* as one major player (27–29). Remarkably, GSH depletion significantly decreased the expression of these key fibrogenic factors under both chow and HFD conditions (Figure 3, A and B). Liver fibrosis is often accompanied by chronic inflammation, which further contributes to disease progression. Indeed, under HFD, GSH depletion also significantly decreased expression of *Ccl2* and *Tnfa* (TNF-α), cytokines that promote inflammation and immune cell recruitment (Figure 3B). These results indicate that GSH depletion confers robust antifibrotic and antiinflammatory effects in liver.

Given the cellular heterogeneity of the liver and the complex intercellular crosstalk involved in disease progression (28), we then asked whether GSH depletion–induced suppression of lipogenesis and fibrosis is intrinsic to hepatocytes. To address this, we isolated primary mouse hepatocytes and infected them with adenoviruses expressing *Chac1* or GFP, followed by treatment with BSA or BSA-conjugated fatty acids to mimic chow and HFD conditions, respectively. Consistent with in vivo findings, GSH depletion significantly decreased the expression of genes involved in lipogenesis and fibrosis in hepatocytes under both treatment conditions (Figure 3, D and E).

Taken together, these results demonstrate that hepatic GSH depletion induces a functional adaption in the transcriptional program, leading to decreased lipid accumulation and fibrosis in liver, and these effects are predominantly intrinsic to hepatocytes.

### GSH depletion decreases hepatic neutral lipid accumulation under HFD.

**Figure 4:**
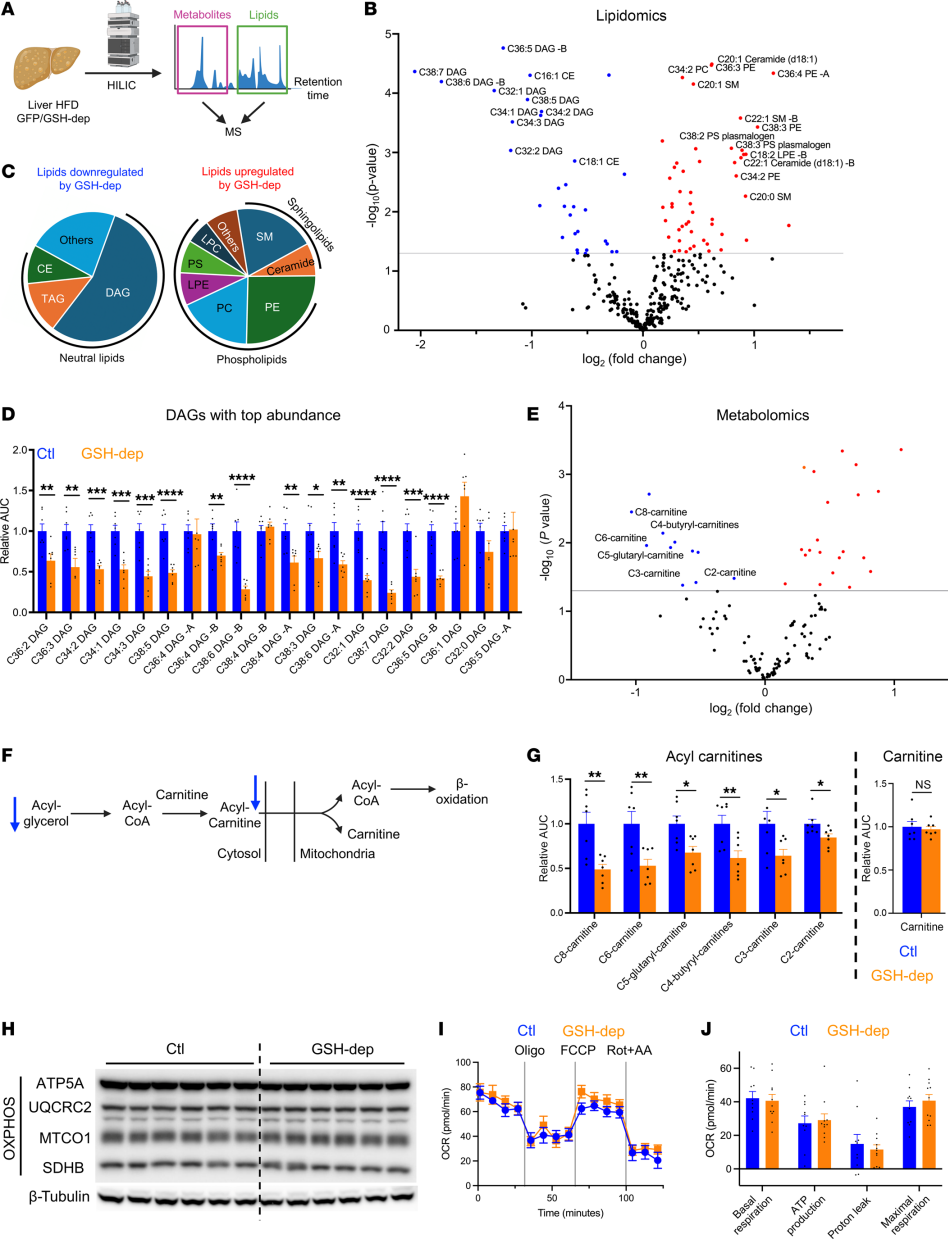
Hepatic lipid profile is remodeled by GSH depletion. (**A**) Metabolite and lipid profiling in mouse livers under HFD. HILIC, hydrophilic interaction liquid chromatography. (**B**) Volcano plot showing lipid alterations in mouse livers following GSH depletion. Red: upregulated lipids; Blue: downregulated lipids. (**C**) Distribution of lipid species regulated by GSH depletion. DAG, diacylglycerol; TAG, triacylglycerol; CE, cholesterol esters; SM, sphingomyelin; PE, phosphatidylethanolamine; PC, phosphatidylcholine; LPE, lysophosphatidylethanolamine; PS, phosphatidylserine; LPC, lysophosphatidylcholines. (**D**) Levels of DAGs in mouse livers. Lipids with -A and -B mean detected isomers with 2 peaks (*n* = 7). (**E**) Volcano plot showing metabolite alterations in mouse livers following GSH depletion. Red: upregulated metabolites; Blue: downregulated metabolites. (**F**) Metabolism of acylglycerols and carnitine shuttle in cytosol and mitochondria. (**G**) Levels of acyl-carnitines and free carnitine in mouse livers measured by metabolite profiling. (**H**) Protein levels of OXPHOS complexes in livers of mice under HFD. The β-tubulin loading control shown is identical to that in Figure 2O, as the same protein lysates were analyzed. (**I**) Oxygen consumption rate of HepG2 hepatocytes measured using the Agilent Seahorse XFe24 Analyzer under sequential treatment with oligomycin (Oligo), carbonyl cyanide *p*-trifluoromethoxyphenylhydrazone (FCCP), and rotenone plus antimycin A (Rot+AA). (**J**) Quantification of basal respiration, ATP-linked respiration, proton leak, and maximal respiration derived from **I**. Mice were fed an HFD for 2 weeks before infection with AAV and were sacrificed 8 weeks later. *P < 0.05, **P < 0.01, ***P < 0.001, ****P < 0.0001.

To elucidate the mechanisms by which GSH depletion protects against the development of MASLD, we performed untargeted lipidomic and metabolomic profiling on liver tissues from HFD-fed mice (Figure 4A). Lipidomic analysis revealed that GSH depletion significantly decreased levels of neutral lipids, including diacylglycerols (DAGs), triacylglycerols (TAGs), and cholesterol esters (CEs), while GSH depletion increased levels of sphingolipids and phospholipids (Figure 4, B and C, and Supplemental Figure 3, A–C), many of which have been shown to improve hepatic steatosis (26). Interestingly, among the decreased neutral lipid species, DAGs represented the most prominently decreased species (Figure 4C). It has been proposed that DAG accumulation is an important driver of hepatic/systemic insulin resistance and MASLD progression (30–32). Consistent with this, the majority of detected DAG species were significantly lower in livers of GSH-depleted mice under HFD conditions (Figure 4D).

To further assess whether decreased accumulation of neutral lipids was driven by increased fatty acid utilization, we examined metabolites in this pathway. In this process, acyl-glycerol is converted to acyl-carnitine, followed by mitochondrial transport and β-oxidation. In line with decreased expression of key enzymes involving β-oxidation (Figure 3, A and B), metabolomic analysis demonstrated significantly decreased levels of multiple acyl-carnitine species, without alterations in free carnitine levels (Figure 4, E–G). Furthermore, GSH depletion did not change protein levels of oxidative phosphorylation (OXPHOS) complexes in liver under HFD (Figure 4H), and it did not alter mitochondrial respiration in hepatocytes (Figure 4, I and J). Taken together, these data indicate that the decreased accumulation of hepatic neutral lipids in GSH-depleted mice is primarily attributed to decreased lipogenesis, rather than enhanced fatty acid mobilization.

### GSH depletion selectively oxidizes and destabilizes FASN.

GSH plays a critical role in maintaining protein cysteine residues in a reduced state, and oxidized proteins are typically functionally impaired and prone to degradation (33, 34). Thus, we hypothesized that GSH depletion enhances the oxidation of key lipogenic factors, rendering them dysfunctional and targeting them for degradation.

**Figure 5:**
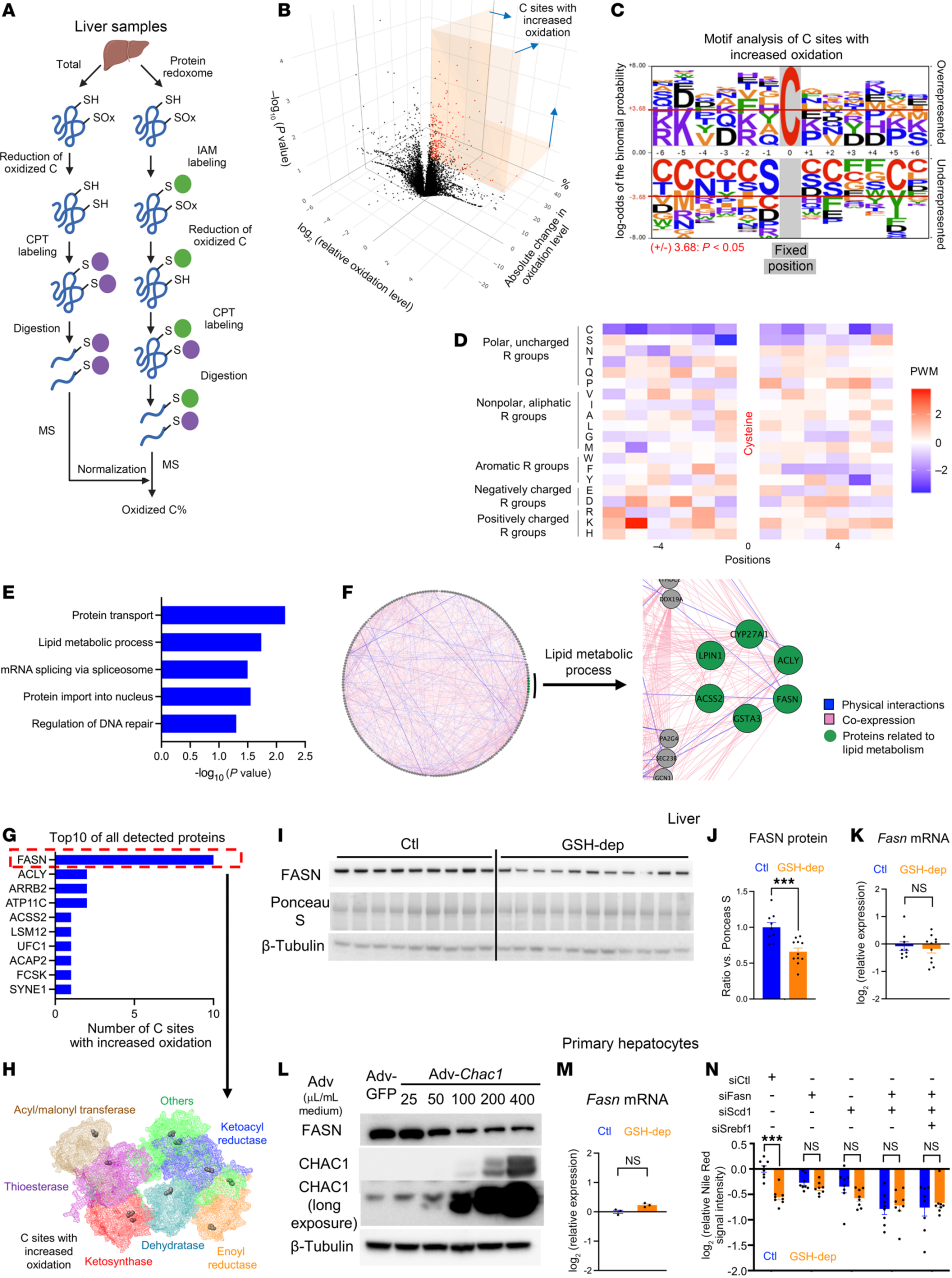
Selective protein oxidation by GSH depletion. (**A**) Protein redoxome analysis on mouse livers. SH, thiol; SOx, oxidized thiol; IAM, iodoacetamide; CPT, cysteine-reactive phosphate tags. (**B**) 3D volcano plot showing cysteines with differential levels of oxidation following GSH depletion. Red: cysteines with increased oxidation. Cysteines with significantly increased oxidation were defined as those with a *P* < 0.05, with a relative oxidation increase of at least 25%, and falling within the top 15% of all sites ranked by the absolute increase of oxidation level. (**C**) Motif analysis of cysteines with increased oxidation. Cysteine is fixed at the position 0. Over- and underrepresented motifs were identified using pLOGO (https://plogo.uconn.edu/). (**D**) Position weight matrix of amino acids in overrepresented motifs surrounding cysteine sites with increased oxidation. (**E**) Pathways enriched in proteins containing cysteines with increased oxidation. Pathways were identified using DAVID GO analysis (https://davidbioinformatics.nih.gov/) , with the top 5 pathways ranked by *P* value displayed. (**F**) Interactome of proteins containing cysteines with increased oxidation generated using GeneMANIA. (**G**) Rank of detected proteins by the number of cysteines with increased oxidation (top 10 shown). (**H**) Predicted structure of mouse FASN (AF-P19096-F1-v4). Cysteines with increased oxidation were labeled black. (**I**) Immunoblot analysis of FASN in mouse livers. The β-tubulin loading control is identical to Figure 1C, as the same protein lysates were analyzed. (**J**) Quantification of immunoblots. (**K**) *Fasn* mRNA levels in mouse liver. (**L**) Protein levels of FASN in primary hepatocytes following *Chac1* overexpression using Adv. Cells were harvested 3 days postinfection. (**M**) *Fasn* mRNA levels in primary hepatocytes (Adv: 400 μL/mL medium; 3 days postinfection). (**N**) Lipid accumulation in primary hepatocytes. Cells were treated with siRNAs and palmitic acid (0.25 mM) 12 hours after infection with Adv overexpressing GFP or *Chac1*. Nile red staining was performed 3 days postinfection. ***P < 0.001.

To systemically assess reversible protein cysteine oxidation, we performed protein redoxome analysis (35) on liver samples from control and GSH-depleted mice. Using redox-specific cysteine-labeling strategies coupled with mass spectrometry, we quantified reversible oxidation states at individual cysteine residues (Figure 5A). Surprisingly, GSH depletion did not lead to global increase in protein oxidation but instead selectively enhanced oxidation at specific cysteine sites (Figure 5B and Supplemental Figure 4, A–D). A total of 11,611 cysteine sites were detected, of which only 1.8% exhibited increased levels of oxidation following GSH depletion. Motif analysis of the amino acid sequences flanking the cysteines with higher oxidation levels revealed a higher frequency of positively charged residues at positions -5 and -6 (Figure 5, C and D), indicating sequence-specific susceptibility to oxidation by GSH depletion. Gene Ontology (GO) enrichment analysis of proteins harboring cysteines that exhibit increased oxidation indicated a strong association with pathways involved in protein transport and lipid metabolism (Figure 5E). GeneMANIA interactome analysis (36) showed that lipid metabolism-related proteins form extensive physical interactions between themselves and with other proteins harboring oxidized cysteines (Figure 5F and Supplemental Figure 4E), indicating the potential for functional coregulation within a redox-sensitive lipid metabolic protein network.

To identify the most affected targets, we ranked all detected proteins based on the number of cysteine sites exhibiting increased oxidation in GSH-depleted livers. FASN emerged as the top candidate, with 10 cysteine sites showing increased oxidation levels, far exceeding other proteins, which had at most 2 sites (Figure 5G). FASN, a key enzyme in lipogenesis, catalyzes the synthesis of long-chain fatty acids, which are then used to synthesize neutral lipids, such as acylglycerols and CEs (37, 38). FASN has a complex structure composed of 2 distinct regions (the condensing region and the modifying region), each of which contains multiple catalytic domains (39). The condensing region includes regions with ketosynthase and acyl/malonyl transferase activities, while the modifying region includes dehydratase, enoyl reductase, ketoacyl reductase, and thioesterase domains (39, 40) (Figure 5H). Interestingly, these 10 cysteines with higher levels of oxidation were distributed across all domains of FASN (Figure 5H and Supplemental Figure 4F). Given that oxidized proteins are prone to degradation (34), we hypothesized GSH depletion could result in a lower level of FASN protein in liver. Indeed, immunoblot analysis revealed significantly decreased protein levels of FASN in livers from GSH-depleted mice (Figure 5, I and J), without decreased levels of *Fasn* mRNA (Figure 5K). These data indicate FASN was downregulated at the posttranslational level by oxidation-induced protein degradation due to GSH depletion.

We then asked whether GSH depletion–mediated FASN downregulation is intrinsic to hepatocytes. To test this, we infected mouse primary hepatocytes with adenoviruses expressing either *Chac1* or GFP. Immunoblot analysis showed FASN protein levels were substantially decreased in a dose-dependent manner with *Chac1* overexpression (Figure 5L), indicating this effect by GSH depletion is intrinsic to hepatocytes. Again, the *Fasn* mRNA levels remained unchanged (Figure 5M), reinforcing the posttranslational downregulation of FASN by GSH depletion.

Previous studies have suggested that *Chac1* may also antagonize Notch signaling, especially in the process of neurogenesis (41, 42). To examine whether the decreased FASN levels could result from NOTCH inhibition, we assessed the level of Notch signaling by identifying the levels of NOTCH1, NOTCH2, and their most direct downstream effector, HES1 (43), in GSH-depleted livers. However, no significant changes were observed in these proteins (Supplemental Figure 4G), suggesting that the suppression of lipogenesis via FASN oxidation is independent of Notch signaling.

In addition to downregulating FASN at the posttranslational level, GSH depletion also decreased the key lipogenic genes at the transcriptional level, most notably *Scd1* and *Srebf1* (Figure 3, A–C). Interestingly, knockdown of *Fasn*, *Scd1*, or the combination of both together with *Srebf1* significantly decreased lipid accumulation in primary hepatocytes to a degree comparable to that observed upon GSH depletion (Figure 5N). Notably, GSH depletion did not further decrease lipid accumulation in these knockdown conditions, indicating a lack of additive effect. Thus, these data indicate that *Fasn* and *Scd1* may act within the same lipogenic pathway downstream of GSH depletion and that inhibition of either enzyme is sufficient to occlude the lipid-lowering effect.

Collectively, these data indicate that GSH depletion selectively promotes oxidation and degradation of FASN in hepatocytes. Combined with transcriptional inhibition of lipogenic genes, lipogenesis is thereby inhibited through both posttranslational and transcriptional mechanisms.

### GSH depletion protects from methionine/choline-deficient diet–induced liver injury.

To determine whether GSH depletion can also exert protective effects in a primarily MASH model, we overexpressed *Chac1* in mouse liver using AAV8 followed by challenging mice with methionine/choline-deficient diets (MCDs) to induce MASH. In this context, GSH-depleted and control mice showed comparable body weights (Supplemental Figure 5A). GSH depletion modestly increased the weight of BAT, but the weights of liver and other metabolic organs, such as eWAT, iWAT, and muscle, were unchanged (Supplemental Figure 5, B–F). Fasting glucose levels were also similar between these 2 groups (Supplemental Figure 5G). Notably, however, GSH depletion significantly decreased circulating ALT levels (Supplemental Figure 5H), indicating protective effects against MCD-induced liver damage.

### Chac1 expression is suppressed during feeding and MASLD/MASH.

**Figure 6:**
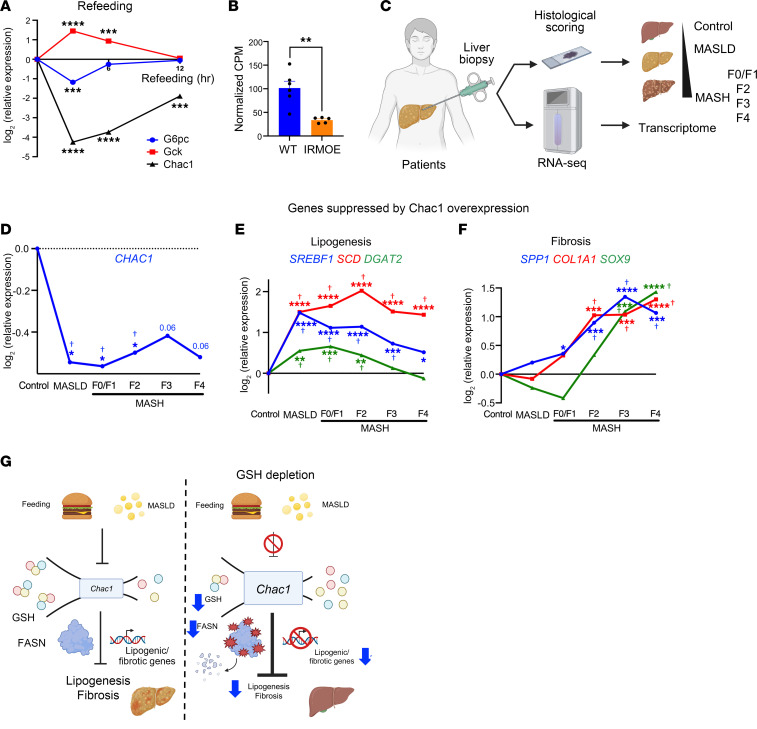
Transcriptional regulation of *Chac1* in metabolic transitions and MASLD. (**A**) Relative expression of G6pc, Gck, and *Chac1* in livers under fasting or after refeeding. Expression was assessed by transcriptomics (RNA-seq). Raw data were from National Center for Biotechnology Information (NCBI) Gene Expression Omnibus (GEO) GSE137385. (**B**) Relative expression of *Chac1* in skeletal muscles of WT or muscle-specific insulin receptor overexpression (IRMOE) mice. The RNA-seq data were from accession number GSE149662. CPM, counts per million. (**C**) Schematic showing liver biopsy from patients with MASLD/MASH followed by histological scoring and transcriptomic analysis (RNA-seq). Raw data were from GSE135251. (**D**) Relative expression of CHAC1 in liver biopsies from MASLD/MASH. (**E** and **F**) Relative expression of key genes suppressed by hepatic *Chac1* overexpression involved in lipogenesis (**E**) and fibrosis (**F**) in liver biopsies from MASLD/MASH. (**G**) Schematic showing hepatic GSH depletion by *Chac1* overexpression protects from MASLD through selective protein oxidation and transcriptional regulation. Significance for transcriptomic analysis was determined using DESeq2, unless otherwise specified. For **D**–**F**, Wald’s test *P* value is indicated as *P < 0.05, **P < 0.01, ***P < 0.001, ****P < 0.0001; Benjamini-Hochberg *P* value is indicated as ^†^*P* < 0.1.

Given that *Chac1* overexpression–mediated glutathione depletion substantially suppresses lipogenesis and mitigates MASLD/MASH progression, we asked whether *Chac1* expression is regulated during normal metabolic transitions or in liver disease states. To this end, we analyzed transcriptomics from mouse livers under fasting conditions and at multiple time points following refeeding (44). Clustering analysis of the changes of expression revealed distinct transcriptional kinetics among genes during refeeding (Supplemental Figure 6A). Notably, *Chac1* expression was significantly downregulated by approximately 95% as early as 3 hours after refeeding, and this suppression persisted for at least 12 hours (Figure 6A). The magnitude of *Chac1* downregulation exceeded that of canonical refeeding-responsive genes controlling hepatic glucose metabolism, such as the downregulation of *G6pc* or upregulation of *Gck* (Figure 6A), indicating robust transcriptional suppression of *Chac1* during feeding. Since insulin sensitivity plays a central role in transcriptional adaptation to metabolic cues, we next examined whether *Chac1* expression is regulated by changes in insulin sensitivity. In mice overexpressing the insulin receptor in skeletal muscle (Supplemental Figure 6B) (45), transcriptomic profiling of muscle revealed a significant downregulation of *Chac1* (Figure 6B and Supplemental Figure 6C). Thus, these data demonstrate that *Chac1* expression is highly regulated corresponding to feeding and changes of insulin sensitivity.

Finally, we asked whether the expression of *Chac1* is altered during the development of human MASLD/MASH. In the dataset from Govaere et al., more than 200 individual liver biopsies from patients were histologically scored to determine MASLD/MASH stage, followed by RNA-seq for transcriptomic profiling (46) (Figure 6C). Differential gene expression analysis revealed a continuous trajectory of transcriptional changes with stage of disease progression from MASLD to advanced MASH (Supplemental Figure 7, A and B), indicating substantial transcriptional remodeling during MASLD/MASH progression. Likewise, GO analysis demonstrated enrichment of extracellular matrix remodeling and fibrotic pathways, consistent with disease progression (Supplemental Figure 8). Interestingly, *Chac1* was downregulated across the entire disease spectrum, beginning in early MASLD and remaining suppressed through progression to MASH with advanced fibrosis (Figure 6D). In contrast, the key lipogenic and fibrogenic genes that are suppressed by *Chac1* overexpression–induced hepatic GSH depletion, including *Srebf1*, *Scd*, *Dgat2*, *Spp1*, *Col1a1*, and *Sox9*, were upregulated during disease progression (Figure 6, E and F).

Taken together, these data demonstrate that loss of *Chac1* expression is associated with metabolic dysregulation and fibrosis in MASLD/MASH and that restoring *Chac1* expression and the resulting GSH depletion may represent a potentially novel therapeutic strategy for halting MASLD/MASH progression.

## Discussion

GSH supplementation has been proposed as a potential therapy to ameliorate MASLD/MASH. This is largely based on the widely accepted assumption that hepatic glutathione depletion leads to general oxidative stress, thereby promoting the development of MASLD/MASH. To define the physiological role of GSH depletion in the development of MASLD, we leveraged liver-specific overexpression of *Chac1*, a recently identified cytosolic GSH-degrading enzyme, to achieve efficient and targeted hepatic intracellular GSH depletion. Contrary to the conventional view that GSH depletion exacerbates MASLD and induces widespread protein oxidation, our findings demonstrate that GSH depletion substantially protects against MASLD. This occurs through selective oxidation on FASN and transcriptional inhibition on lipogenic genes, leading to decreased lipogenesis and fibrosis. Thus, this study revises the conventional doctrine of GSH function in protein redox regulation and liver pathophysiology, indicating that hepatic GSH depletion by *Chac1* overexpression may represent a potentially novel therapeutic approach for MASLD (Figure 6G).

We show GSH depletion significantly decreased the level of *Srebf1* (protein: SREBP-1c), a critical regulator of lipogenic gene transcription (Figure 3). Although most *Srebf1* downstream genes were downregulated at the transcriptional level, some were not. The differential regulation on these genes may be due to several factors. Initially, it has been shown that SREBP binds to sterol regulatory elements (SREs), and the original SRE sequence was defined as 5′-ATCACCCAC-3′ (47). In addition to the SRE, later it was shown that SREBP-1c can bind to E-boxes (CAXXTG) (48). This dual binding specificity of SREBP-1c enables it to bind to different lipogenic gene promoters with different affinity and stability. In addition, although the SREBP-1c–binding sequences in these promoters share some common bases, the exact sequences vary hugely (49), which may also contribute to different binding affinity and different regulatory pattern.

Inhibiting lipogenesis has long been proposed as a potential therapeutic strategy for ameliorating MASLD. Thus, several key lipogenic genes have been targeted in liver followed by extensive examination of metabolic effects, including *Fasn*, *Scd1*, and *Srebf1*. Recently, it has been shown that hepatic *Fasn* KO substantially ameliorated hepatic steatosis in multiple MASLD models, such as *ob/ob* and *Mc4r*-KO (50). In addition, *Fasn* inhibition using small molecules substantially reduced hepatic steatosis in mice fed with Western diets and human primary liver microtissues (51). Similarly, *Scd1* KO ameliorated hepatic steatosis under HFD. *Scd1* deficiency has been shown to decrease lipogenesis and to increase fatty acid oxidation (52). However, in hepatic GSH-depleted mice, although decreased lipogenesis was observed, we did not observe increased fatty acid oxidation. This could be due to different levels of *Scd1* deficiency by KO versus knockdown. With regard to *Srebf1*, its deficiency ameliorated fatty liver diseases in *ob/ob* models with decreased expression levels of lipogenic genes (53). Later, it was shown that transient knockdown of *Srebf1* using antisense oligonucleotide in diet-induced obese mice decreased the lipogenic gene expression and ameliorated hepatic steatosis (54). In addition, decreasing nuclear *Srebf1* levels by deletion of *Scap*, which is necessary to generate nuclear isoforms of *Srebf1*, protected against hepatic steatosis in *ob/ob* mice and mice under HFDs (55). Together, these studies show that inhibition of lipogenic pathways protects from MASLD in multiple models with decreased levels of lipogenic genes, and that GSH depletion through *Chac1* overexpression recapitulates similar protective effects by broadly downregulating lipogenic genes. This study demonstrates that manipulating hepatic GSH levels is an effective method to efficiently target lipogenic pathways.

Although GSH depletion protects against liver injury in both HFD and MCD models (Figure 2 and Supplemental Figure 5), the magnitude of protection is much greater in HFD. We interpret this difference as reflecting distinct disease mechanisms underlying MASLD and MASH. In HFD, hepatocytes are exposed to excessive fat, leading to upregulation of lipogenesis and acylglycerol storage. In contrast, MCD leads to severe deficiency of methionine and choline, disrupting phosphatidylcholine synthesis, VLDL secretion, and mitochondrial respiration, which rapidly induces inflammation and liver damage. Consistent with these mechanistic differences, increased expression of key lipogenic genes, such as *Fasn* and *Srebf1*, was prominent in MASLD but not MASH, both in murine models and in patient samples (56). Because GSH depletion primarily exerts its protective effects by suppressing lipogenesis, its benefit is expected to be greater in HFD-driven MASLD than in MCD-driven MASH. These observations underscore an important therapeutic implication for MASLD, showing that the timing and disease stage may be critical for maximizing the protective effects of GSH depletion, with the greatest benefit likely occurring during early, lipogenesis-driven phases of MASLD progression.

In addition to *Chac1* overexpression, GSH depletion can be achieved by inhibiting GSH synthesis using genetic or pharmacological strategies targeting glutamate-cysteine ligase catalytic subunit (*Gclc*) or modifier subunit (*Gclm*) (11–13, 57). Interestingly, although *Gclc* or *Gclm* deficiency also decreases hepatic lipid accumulation accompanied with downregulated expression levels of lipogenic genes, *Chac1* overexpression exhibits distinct phenotypic differences in liver from these models. Hepatic *Gclc* KO has been shown to increase levels of serum ALT and AST, suggesting increased liver injury, whereas *Chac1* overexpression leads to decreased levels of ALT and AST, indicating a protective effect. The opposing effects on liver protection in *Gclc* KO versus *Chac1* overexpression may also reflect differences in the severity of GSH depletion. In *Gclc*-KO liver, the GSH level is less than 5%, but in *Chac1*-overexpression mice, the liver GSH level is approximately 15%, which is sufficient to maintain normal liver function and mitochondrial respiration (14). At the molecular level, hepatic *Gclc* or *Gclm* KO triggers a robust activation of *Nrf2* and its target genes in liver (12, 13), indicating increased oxidative stress response, whereas *Nrf2* level is not increased in livers with *Chac1* overexpression. These distinctions highlight that the mode of GSH depletion, i.e., via acute blockade of synthesis versus more gradual enhanced degradation, can differentially influence the oxidative stress response, secondary transcriptional programs, and systemic physiology. Acute complete depletion may rapidly amplify oxidative stress and cell injury, whereas more gradual depletion appears to allow for more selective redox remodeling without overwhelming stress responses, although further studies including comprehensive transcriptomic and epigenomic analyses will be necessary to dissect the differential regulatory networks altered by different modes of GSH depletion. In addition, the differences in liver injury observed between *Gclc*/*Gclm* deficiency and *Chac1* overexpression may, in part, reflect the developmental effects in the KO mice. An important advantage of inducing GSH depletion in adult mice through *Chac1* overexpression is that it avoids these developmental confounders.

Beyond identifying a potential therapeutic target, our study prompts a fundamental reassessment of the role of GSH in protein oxidation and liver pathophysiology. Although oxidative stress and increased lipogenesis are well-recognized contributors to MASLD development, the assumed necessity of GSH in broadly mitigating protein oxidative damage has been insufficiently scrutinized. By directly analyzing the protein redoxome, our data reveal that GSH depletion does not indiscriminately oxidize proteins but instead induces highly selective oxidation events, particularly affecting FASN. The mechanisms underlying this selectivity remain to be fully elucidated but may involve localized interactions between specific proteins and GSH-degrading enzymes, resulting in enzyme-centered sub-compartmentalized GSH depletion. Alternatively, selective exposure of reactive cysteines, especially those located within protein functional pockets or solvent-accessible regions, may confer differential susceptibility to oxidation (58–60). Future work will need to systematically dissect the structural and spatial determinants of GSH-mediated redox selectivity.

Our findings also highlight the need to revisit the broader antioxidant landscape in redox biology. Other nonenzymatic antioxidant systems, such as vitamins C and E, may also display selective, context-dependent effects rather than exerting global protein reduction, suggesting that a deeper understanding of antioxidant specificity is critical for developing targeted therapies (61, 62).

Finally, the use of tissue-specific overexpression of GSH-degrading enzymes, as exemplified by liver-specific *Chac1* overexpression, establishes a conceptual framework for dissecting antioxidant functions in a tissue- and cell type–specific manner. While circulating GSH has been proposed to play important systemic roles (1), GSH is distributed across virtually all tissues and cell types (12); its biological functions are likely to vary depending on cellular metabolic activity and microenvironmental factors. Indeed, global GSH depletion in mice has been shown to cause rapid weight loss and death within 2 weeks, whereas liver-specific GSH depletion does not result in such systemic toxicity, despite the liver being the primary site of GSH synthesis (12). Tissue-specific manipulation of GSH degradation therefore provides a powerful tool to distinguish local versus systemic antioxidant effects and to uncover therapeutic opportunities tailored to organ-specific pathologies.

In conclusion, our study revises the traditional view of GSH as a global protective antioxidant against protein oxidation under physiological condition and demonstrates that GSH depletion through enhanced degradation leads to selective protein oxidation. More importantly, our study shows that manipulating protein redox balance through GSH depletion in liver confers unexpected and substantial protection against MASLD. These insights create a paradigm for therapeutic strategies targeting lipogenesis and GSH-mediated protein redox homeostasis through controlled GSH degradation in liver disease.

## Methods

### Sex as a biological variable.

Our study examined male mice because male animals exhibited less variability in phenotypes related to diet-induced MASLD. While female mice were not examined in the current study, the core pathways investigated, including hepatic lipogenesis and glutathione-mediated redox regulation, are largely conserved across sexes. Therefore, the findings are expected to be broadly relevant to both male and female physiology, although future studies will be required to directly assess sex-specific effects.

### Animal care and use.

Male mice were used for studies unless indicated. Liver-specific *Chac1* and GFP overexpression mice were generated through retro-orbital injections of AAV8 (4 × 10^10^ viral particles per mouse) expressing *Chac1* or GFP controlled by human TBG promoter. Physiological experiments were performed between 4 and 8 weeks after AAV infection, and mice were sacrificed 8 weeks after AAV infection. All mice were housed at 22°C on a 12-hour light/12-hour dark cycle with ad libitum access to food and water. Lights were turned on at 7:00 am. Mice were on C57BL/6 background (The Jackson Laboratory), fed either chow diet (Mouse Diet 9F, PharmaServ) or HFD (Research Diets, D12492). The caloric composition of the normal chow diet consisted of 23% protein, 21.6% fat, and 55.4% carbohydrates, while the HFD contained 20% protein, 60% fat, and 20% carbohydrates.

### Metabolomics and lipidomics.

Polar and nonpolar lipids from liver tissues were profiled with the use of a Vanquish UHPLC system coupled to an Orbitrap IQ-X mass spectrometer (Thermo Fisher Scientific). Lipids were extracted from liver homogenization (10 μL) with the use of 190 μL isopropanol containing 1,2-didodecanoyl-*sn*-glycero-3-phosphocholine as an internal standard (Avanti Polar Lipids). After centrifugation (20 minutes; 18,000*g*; 4°C), supernatants (10 μL) were injected directly onto a 1.7 μm 100 × 2.1 mm ACQUITY BEH C8 column (Waters). The column was eluted isocratically at a flow rate of 450 μL/min for 1 minute at 80% mobile-phase A (10 mM NH_4_Ac/methanol/acetic acid 95:5:0.1, by volume), followed by a linear gradient to 80% mobile-phase B (methanol/acetic acid; 99.9/0.1 v/v) over 2 minutes, a linear gradient to 100% mobile-phase B over 7 minutes, and then 3 minutes at 100% mobile-phase B. Mass spectrometry analyses were carried out with the use of electrospray ionization in the positive-ion mode with the use of full-scan analysis over *m*/*z* 200–1,100 at 120,000× resolution and a 3 Hz data acquisition rate. Additional mass spectrometry settings were as follows: ion spray voltage, 3.0 kV; capillary temperature, 300°C; probe heater temperature, 300°C; sheath gas, 50 arbitrary units; auxiliary gas, 15 arbitrary units; and S-lens radiofrequency level, 60%. Raw data were processed with Progenesis QI version 1.0.5165.27075 (NonLinear Dynamics) for feature alignment, nontargeted signal detection, and signal integration. Targeted processing of a subset of lipids was conducted with TraceFinder version 3.2 (Thermo Fisher Scientific). Lipids were denoted by headgroup and total acyl carbon and double-bond content.

To measure organic acids and other intermediary metabolites in negative ionization or amide mode, chromatography was performed on an Agilent 1290 Infinity LC system equipped with a Waters XBridge Amide column, coupled to an Agilent 6490 triple-quadrupole mass spectrometer. Metabolite transitions were assayed using a dynamic multiple reaction monitoring system. LC-MS data were analyzed with Agilent Masshunter QQQ Quantitative analysis software.

Levels of GSH and GSSG in livers of mice in Figure 2A and Supplemental Figure 1, C and D were measured using Glutathione Assay Kit (Cayman Chemical, 703002).

### Virus production.

For the AAV, AAV-TBG-*Chac1* was generated from AAV-TBG-GFP (Addgene, 105535) by replacing the GFP sequence with “*Chac1*-myc” subcloned from pcDNA3.1-*Chac1*-myc. The details of AAV production have been previously described (63). Briefly, AAV was packaged and produced in HEK293T cells (ATCC). Cells were transfected with AAV shuttle vector (7 μg per 15 cm dish), Rep/Cap plasmids (7 μg per 15 cm dish) and Δ F6 helper plasmids (20 μg per 15 cm dish) using polyethyleneimine. At 72 hours posttransfection, cells were dislodged and harvested. AAV was then purified through iodixanol gradient ultracentrifugation (for 2 h 40 min at ~381,600 × *g* [rotor: 70Ti, Beckman Coulter] at 14°C) and titered by qPCR.

For Adv, *Chac1*-myc was subcloned into the pACAd5-IRES-GFP vector. Empty pACAd5-IRES-GFP vector was utilized as the control. The vector was linearized with PacI and was cotransfected with pACAd-9.2-100 into 293AD cells (Cell Biolabs, AD-100) according to the manufacturer’s protocol (Cell Biolabs, VPK-254). Crude 293AD cell lysates were harvested 10 days posttransfection and were used to infect primary hepatocytes.

### Insulin tolerance test.

Insulin tolerance tests were performed in 3-hour–fasted mice by intraperitoneal injection of 1.4 mU insulin/kg body mass on chow diet or 2 mU insulin/kg body mass on HFD. Blood glucose levels were measured every 15 to 60 minutes using an Infinity glucose meter (US Diagnostics).

### Comprehensive metabolic phenotyping.

Mice were placed in a Comprehensive Lab Animal Monitoring System for metabolic phenotyping 6–7 weeks after AAV infection. Prior to data collection, mice were acclimated in individual chambers for 24 hours. At the start of the experiment, each mouse was placed in a dedicated chamber for continuous monitoring. The oxygen consumption (VO_2_) and carbon dioxide production (VCO_2_) were measured after calibration. Throughout the experiment, mice had ad libitum access to food and water. Metabolic parameters including energy expenditure, RER, locomotor activity, and cumulative food intake were continuously recorded and analyzed.

### Nonlabeled hyperinsulinemic-euglycemic clamp.

Mice underwent surgical implantation of an indwelling catheter into the right internal jugular vein under ketamine/xylazine anesthesia. Following a 4- to 5-day recovery period, mice were fasted overnight and placed in rat-sized restrainers for acclimation. Insulin was then continuously perfused using a dose of 15 pmol/kg/min. Blood samples were collected from the tail tip every 10–20 minutes for the immediate measurement of blood glucose, and a 20% glucose solution was infused as needed to maintain blood glucose levels within the euglycemic range. At the end of the clamp, mice were euthanized. Tissues were immediately harvested, snap-frozen in liquid nitrogen, and stored at –80°C for subsequent analysis. All clamp experiments were performed at the National Mouse Metabolic Phenotyping Center at UMass Medical School.

### Analysis of tissue histology and liver injury.

H&E and Sirius red staining were performed by Rodent Histopathology Core at Harvard Medical School. Triglyceride levels in the liver were measured using Pointe Scientific Triglycerides Liquid Reagents (T7532). Serum ALT and AST levels were measured using kits from Stanbio (catalog 2930-430, 2920-430).

### Protein redoxome and interactome analysis.

The details of the protocol have been previously described (35). Liver samples were lysed rapidly in 20% ice-cold trichloroacetic acid (TCA), and the resulting lysate from each sample was split into 2 identical half-samples containing approximately 200 μg proteins each. The pellet was then washed with 20% TCA, 10% TCA, and 5% TCA twice. One half-sample was resuspended in 100 mM HEPES pH 8.5, 2% SDS, 1 mM EDTA, 1 mM DTPA, 10 μM neocuproine, and 35 mM IAM for 2 hours at 37°C in the dark, and the other half-sample was treated with labeling buffer 100 mM HEPES, 2% SDS, 1 mM EDTA, 1 mM DTPA, 10 μM neocuproine, and 35 mM CPT. After labeling, proteins in both half-samples were precipitated by methanol and chloroform, and resuspended in the CPT-containing buffer above plus 5 mM TCEP to label reversibly modified cysteines. Proteins were precipitated again and digested with LysC and trypsin in 200 mM EPPS buffer pH 8.0 at a 100:1 substrate-to-enzyme ratio overnight at 37°C. Digests were then labeled by TMT 16-plex reagent following the SL-TMT protocol (64). After combining TMT-labeled peptides, the mixed samples were desalted using a Sep-Pak cartridge (Waters), treated with Lambda phosphatase (Santa Cruz Biotechnology), and desalted again. The High-Select Fe-NTA Phospho-peptide Enrichment Kit (Thermo Fisher Scientific) was then used to enrich CPT-labeled peptides following the manufacturer’s instructions. Samples were then desalted again, and fractionated to 12 fractions using a 57-minute linear gradient from 3% to 32% acetonitrile (ACN) in 10 mM ammonium bicarbonate pH 8.0, at a flow rate of 0.25 mL/min, with an Agilent 1100 HPLC. After lyophilization, these 12 fractions were stage-tipped, reconstituted in a solution containing 5% formic acid (FA) and 5% ACN, and analyzed by LC-MS.

Samples were measured on an Orbitrap Eclipse (Thermo Fisher Scientific) with an Easy-nLC 1200 (Thermo Fisher Scientific) using a 180-minute gradient consisting of 2%–23% ACN, 0.125% FA, at 500 nL/min flow rate. A FAIMSPro (Thermo Fisher Scientific) device for FAIMS separation of precursors (65) was operated with default settings and multiple compensation voltages (–35V/–45V/–55V). Peptides were analyzed with a mass range of *m/z* 400–1,600 using 2-second cycles. MS1 resolution was 120,000, with standard automatic gain control target. Singly charged ions were discarded, and other ions were selected and subjected to fragmentation at 35% normalized collisional energy for MS2 with a dynamic exclusion of 120 seconds. Quantification was performed using multinotch SPS-MS3 (66).

The Comet algorithm (67) was used to search all MS/MS spectra against a database containing sequences of mouse (*Mus musculus*) proteins downloaded from UniProt in 2020 (http://www.uniprot.org) with reversed sequences and common contaminant proteins (e.g., human keratins, trypsin). Peptides were searched using following parameters: 25 ppm precursor mass tolerance; 1.0 Da product ion mass tolerance; fully tryptic digestion; up to 2 missed cleavages and 3 modifications; variable modification: oxidation of methionine (+15.9949), CPT (+221.08169) on cysteines; and static modifications: TMT 16-plex (+304.2071) on lysine and peptide N-terminus. The target-decoy method was employed to control the FDR at 1% for both peptides and proteins (68–70). TMT reporter ion signal-to-noise ratios were used for quantification.

The percentage of oxidation at each cysteine site was quantified by calculating the ratio of the levels of reversibly oxidized cysteine and total cysteine, as determined by CPT labeling and TMT-based MS quantification. Motif analysis of amino acid sequences flanking the cysteine was performed using pLOGO pipeline (71). An absolute log-odds value of 3.68 from the binomial probability corresponds to a statistically significant *P* value. Protein interactome analysis was performed using GeneMANIA (36). The protein interaction network was constructed using Cytoscape 3.10.3.

### Protein extraction and immunoblot analysis.

For immunoblot, tissues were homogenized in RIPA buffer (EMD Millipore) with protease and phosphatase inhibitor cocktail (BioTools). Proteins were separated using SDS-PAGE and transferred to PVDF membrane (MilliporeSigma). Immunoblotting was achieved using the indicated antibodies: phospho-IR Y1152/Y1153 (3024; Cell Signaling Technology [CST]), IR (sc-711), phospho-AKT S473 (4060; CST), phospho-AKT T308 (4056; CST), AKT (4685; CST), phospho-IR T1150 (NBP2-60777; Novus Biologicals), phospho-IRS1 S1097 (2385; CST), phospho-JNK (4668; CST), JNK (9252; CST), CHAC1 (ab76386, Abcam), Vinculin (MAB3574, Sigma), FASN (ab22759, Abcam), SCD1 (2438, CST), total OXPHOS (ab110413, Abcam), NOTCH1 (3608, CST), NOTCH2 (5732, CST), HES1 (sc-166410, Santa Cruz Biotechnology), SPP1 (AF808, R&D Systems), and β-tubulin (2146; CST). Quantification of immunoblots was performed using ImageJ (NIH).

### RNA extraction and qPCR analysis.

RNA was extracted by homogenizing tissues or cells in TRIzol, treating with chloroform, and precipitating in 70% ethanol. cDNA was made using High Capacity cDNA Reverse Transcription Kit (Applied Biosystems, catalog 4368813). qPCR was performed utilizing C1000 Thermal Cycler (Bio-Rad, catalog CFX384). Relative abundance of mRNA was normalized to ribosomal protein 36B4 (*Rplp0*). Primer sequences used are listed here: *Chac1* (F: CTGAATGTGAGGGAAGCCGT, R: CACATAGGCCAGTGCTGTGA); *Rplp0* (F: AGATTCGGGATATGCTGTTGGC, R: TCGGGTCCTAGACCAGTGTTC); *Srebf1* (F: GAGCCATGGATTGCACATTT, R: CTCAGGAGAGTTGGCACCTG); *Scd1* (F: GCTGGAGTACGTCTGGAGGAA, R: TCCCGAAGAGGCAGGTGTAG); *Dgat1* (F: GCTCTGGCATCATACTCCATC, R: CGGTAGGTCAGGTTGTCTGG); *Dgat2* (F: TACTCCAAGCCCATCACCAC, R: CAGTTCACCTCCAGCACCTC); *Cd36* (F: TTAGATGTGGAACCCATAACTGGA, R: TTGACCAATATGTTGACCTGCAG); *Acaca* (F: ATGGGCGGAATGGTCTCTTTC, R: TGGGGACCTTGTCTTCATCAT); *Acly* (F: CAGCCAAGGCAATTTCAGAGC, R: CTCGACGTTTGATTAACTGGTCT); *Elovl6* (F: GAAAAGCAGTTCAACGAGAACG, R: AGATGCCGACCACCAAAGATA); *Acsl1* (F: TGCCAGAGCTGATTGACATTC, R: GGCATACCAGAAGGTGGTGAG); *Gpam* (F: ACAGTTGGCACAATAGACGTTT, R: CCTTCCATTTCAGTGTTGCAGA); *Ldlr* (F: TGACTCAGACGAACAAGGCTG, R: ATCTAGGCAATCTCGGTCTCC); *Cpt1a* (F: GAGAAATACCCTGACTATGTG, R: TGTGAGTCTGTCTCAGGGCTAG); *Acadm* (F: GAAGCCACGAAGTATGCCCT, R: CCTTCATCGCCATTTCTGCG); *Acadl* (F: GTCCGATTGCCAGCTAATGC, R: AGGCAGAAATCGCCAACTCA); *Acadvl* (F: ATGGGAGAAGCAGGCAAACA, R: CTCTGGGTGGACAATCCCTG); *G6pc* (F: GTGTCCAGGACCCACCAATA, R: ACTGTGGGCATCAATCTCCT); *Pck1* (F: TGTCTTCACTGAGGTGCCAG, R: CTGGATGAAGTTTGATGCCC); *Spp1* (F: GCTTGGCTTATGGACTGAGGTC, R: CCTTAGACTCACCGCTCTTCATG); *Col1a1* (F: TTGATCCAGAAGGACCTTGTTTG, R: CCTCAGGGTATTGCTGGACAA); *Tgfb1* (F: AAGTTGGCATGGTAGCCCTT, R: GCCCTGGATACCAACTATTGC); *Ccl2* (F: CCCAATGAGTAGGCTGGAGA, R: TCTGGACCCATTCCTTCTTG); *Sox9* (F: CCACCCACCACTCCCAAAAC, R: GCTGCTCAGTTCACCGATGT); *Tnfa* (F: CCACCACGCTCTTCTGTCTA, R: GTGGGTGAGGAGCACGTAGT); *Fasn* (F: GGTTACACTGTGCTAGGTGTTG, R: TCCAGGCGCATGAGGCTCAGC); *Ddit3* (F: CTGGAAGCCTGGTATGAGGAT, R: CAGGGTCAAGAGTAGTGAAGGT); *Nrf2* (F: CAGCATAGAGCAGGACATGGAG, R: GAACAGCGGTAGTATCAGCCAG); *Keap1* (F: ATCCAGAGAGGAATGAGTGGCG, R: TCAACTGGTCCTGCCCATCGTA); *Sod1* (F: TGGGTTCCACGTCCATCAGT, R: TCTCCAACATGCCTCTCTTCATC); *Nos2* (F: GAGGCCCAGGAGGAGAGAGATCCG, R: TCCATGCAGACAACCTTGGTGTTG).

### Primary hepatocyte isolation.

Mouse livers were first perfused with Krebs-Ringer buffer containing 100 μM EGTA (MilliporeSigma) to chelate calcium, followed by enzymatic digestion using 60 mg of type I collagenase (Thermo Fisher Scientific) dissolved in 50 mL of DMEM/F12. After perfusion, liver tissue was carefully dissociated using sterile fine-tipped forceps, and the resulting cell suspension was filtered through a 100 μm sterile strainer (Fisherbrand, 22-363-549) to remove debris and undigested fragments. Hepatocytes were then pelleted by centrifugation at 50*g* for 3 minutes and resuspended in 10 mL of DMEM. To enrich viable cells, the suspension was mixed with 10 mL of Percoll solution (9:1 ratio of Percoll to 10× PBS) and centrifuged at 700*g* for 10 minutes. The viable cell fraction was then washed twice with PBS and plated at a density of 2.5 × 10^5^–3.5 × 10^5^ cells/mL on collagen-coated plates in DMEM supplemented with 20% FBS. After 4 hours, nonadherent cells were removed, and the culture medium was replaced with Lonza HCM hepatocyte medium (CC-3198) or continued in DMEM with 20% FBS, depending on experimental needs.

### Measurement of oxygen consumption rate.

Oxygen consumption rate (OCR) was measured using the Seahorse XFe24 Analyzer (Agilent Technologies) according to the manufacturer’s instructions. HepG2 hepatocytes were seeded in Seahorse cell culture microplates at an optimized density and allowed to adhere overnight. Prior to the assay, cells were washed and incubated at 37°C in a non-CO_2_ incubator for equilibration.

### siRNA transfection.

siRNAs (synthesized by Horizon Discovery) were transfected using Lipofectamine RNAiMAX (Thermo Fisher Scientific). Cells were seeded to reach approximately 80% confluence at the time of transfection. For each well of a 12-well plate, 25–75 pmol siRNA was transfected using 5 μL Lipofectamine RNAiMAX. For 6- or 24-well plates, siRNA and reagent amounts were adjusted proportionally according to surface area.

### Transcriptomic analysis.

For RNA-seq, paired-end FASTQ files were from NCBI GEO accession GSE137385, GSE149662, and GSE135251 and were assessed for quality using FastQC (v0.11.3). Read alignment quality was evaluated with STAR (v2.7.0) and further inspected using Qualimap (v2.2.1), with all samples passing predefined quality thresholds. Transcript quantification was performed by mapping reads to the mm10 or hg38 cDNA reference using Salmon. Aggregated quality metrics were compiled using MultiQC (v1.5). Samples exhibiting atypical clustering patterns in quality control assessments were excluded from further analysis. All computational analyses were conducted using the Harvard Medical School O2 Cluster for high-performance computing. Differential gene expression analysis was carried out using DESeq2 (v1.34.0) to determine relative abundance of transcripts.

### Statistics.

Data are presented as means ± SEM. Significance was determined using a 2-tailed Student’s *t* test, unless otherwise specified in figure legends. Significance is considered as *P* < 0.05.

### Study approval.

Animal studies were performed according to protocols approved by the Joslin Institutional Animal Care and Use Committee and Animal Welfare Committee.

### Data availability.

All data are included in the Supporting Data Values file.

## Author contributions

XYL, GXW, and CRK conceived the study. XYL, GXW, YY, HX, XS, SZ, RG, and CRK curated data. XYL, GXW, YY, HX, XS, SZ, RG, and CRK performed formal analysis. XYL, GXW, CRK, and HX acquired funding. XYL, GXW, YY, HX, XS, SZ, RG, and CRK investigated. XYL, GXW, HX, XS, SZ, RG, and CRK developed methodology. CRK supervised. XYL, GXW, and CRK wrote the manuscript. XYL, GXW, YY, HX, KO, XS, SZ, RG, and CRK provided resources.

## Conflict of interest

The authors have declared that no conflict of interest exists.

## Funding support

This work is the result of NIH funding, in whole or in part, and is subject to the NIH Public Access Policy. Through acceptance of this federal funding, the NIH has been given a right to make the work publicly available in PubMed Central.

- Predoctoral fellowship from American Heart Association 915183 (to XYL).
- NIH grants F99AG088573 (to XYL), T32DK007260-43 (to GXW), R01DK031036 (to CRK), R01DK1121964 (to CRK), P30DK036836 (to Joslin Diabetes Center), R00AG07346103 (to HX).
- Mary K. Iacocca Professorship (to CRK).
- V Scholar Award (to HX).
- Baxter Foundation Faculty Scholars Award (to HX).

## Supplementary Material

Supplemental data

Unedited blot and gel images

Supporting data values

## Version 1 04/15/2026

Electronic publication
