# Supplementary material for: Hepatic glutathione depletion ameliorates MASLD through selective protein oxidation and inhibition of lipogenesis
Source: J Clin Invest. 2026 Apr 15;136(8):e197556. doi: 10.1172/JCI197556 (PMC13078873; doi:10.1172/JCI197556)
Supplement: Supplemental data [file jci-136-197556-s116.pdf]

Figure S1

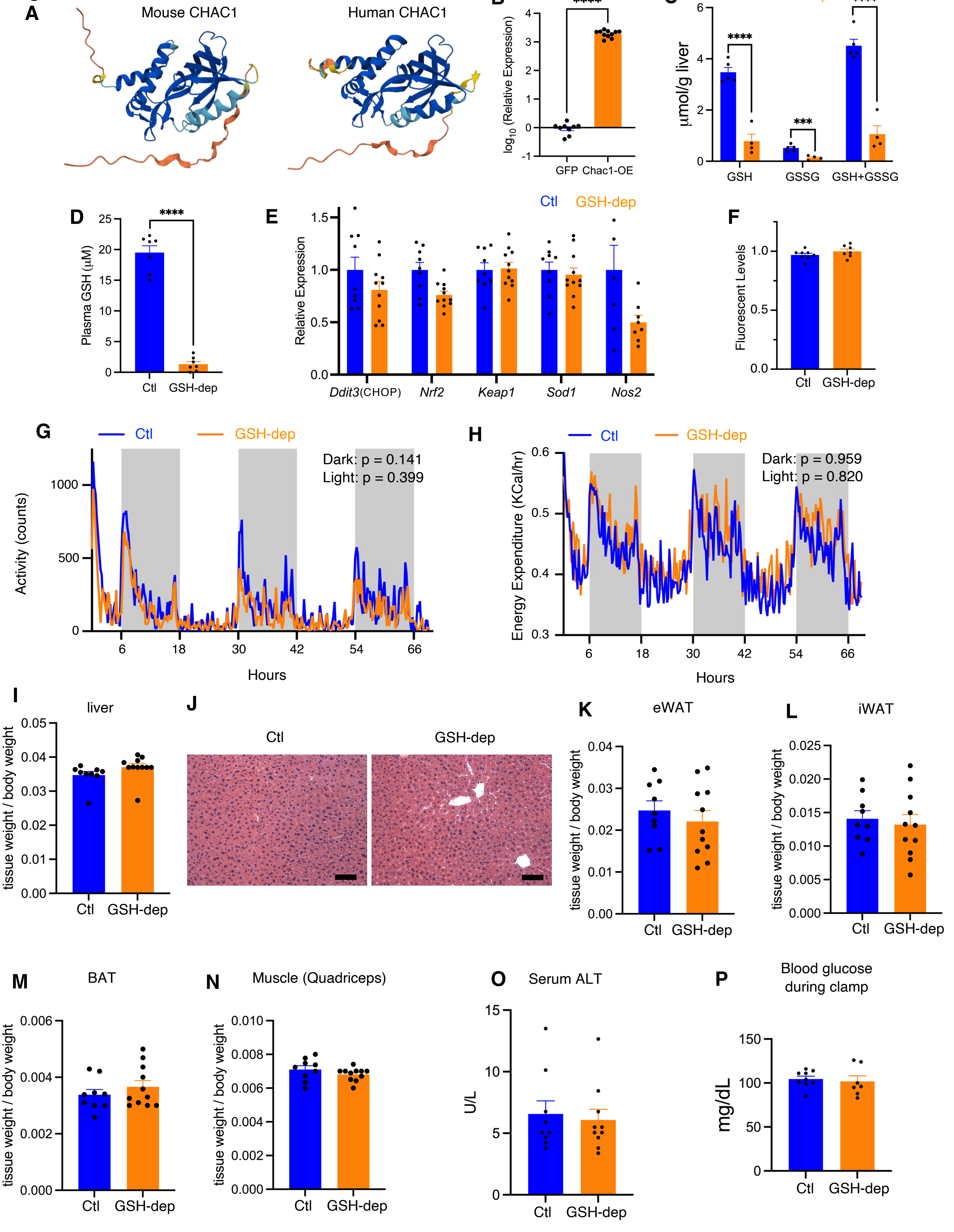

A
Mouse CHAC1
Human CHAC1
B
log10 (Relative Expression)
GFP
Chac1-OE
****
C
Ctl
GSH-dep
µmol/g liver
GSH
GSSG
GSH+GSSG
****
***
D
Plasma GSH (µM)
Ctl
GSH-dep
E
Relative Expression
Ddit3(CHOP)
Nrf2
Keap1
Sod1
Nos2
F
Fluorescent Levels
G
Activity (counts)
Dark: p = 0.141
Light: p = 0.399
Hours
H
Energy Expenditure (KCal/hr)
Dark: p = 0.959
Light: p = 0.820
Hours
I
liver
tissue weight / body weight
J
K
eWAT
L
iWAT
M
BAT
N
Muscle (Quadriceps)
O
Serum ALT
U/L
P
Blood glucose
during clamp
mg/dL

Figure S1. Hepatic GSH depletion through Chac1 overexpression

(A) Predicted protein structures of CHAC1. Mouse: AF-Q8R3J5-F1-v4; Human: AF-Q9BUX1-F1-v4

(B) *Chac1* mRNA levels in mouse livers assessed by qRT-PCR.

(C) Levels of GSH and GSSG in mouse livers measured using Cayman GSH assay kit.

(D) Levels of plasma GSH.

(E) Relative expression levels of genes involved in oxidative stress response in mouse livers. Expression was assessed by qRT-PCR.

(F) Levels of ROS in HepG2 hepatocytes measured using Abcam cellular ROS assay kit. HepG2 cells were maintained under basal conditions without fatty acid treatment during the assay.

(G) Activities of mice across the circadian cycle. (n = 6)

(H) Energy expenditures of mice across the circadian cycle. (n = 6)

(I) Ratio of liver weight to body weight.

(J) H&E staining in the liver. Scale bar: 80 μm.

(K-N) Ratio of tissue weight to body weight.

(O) Circulating levels of ALT in mice.

(P) Levels of blood glucose during hyperinsulinemic-euglycemic clamp.

GSH-dep: GSH depletion.

Figure S2

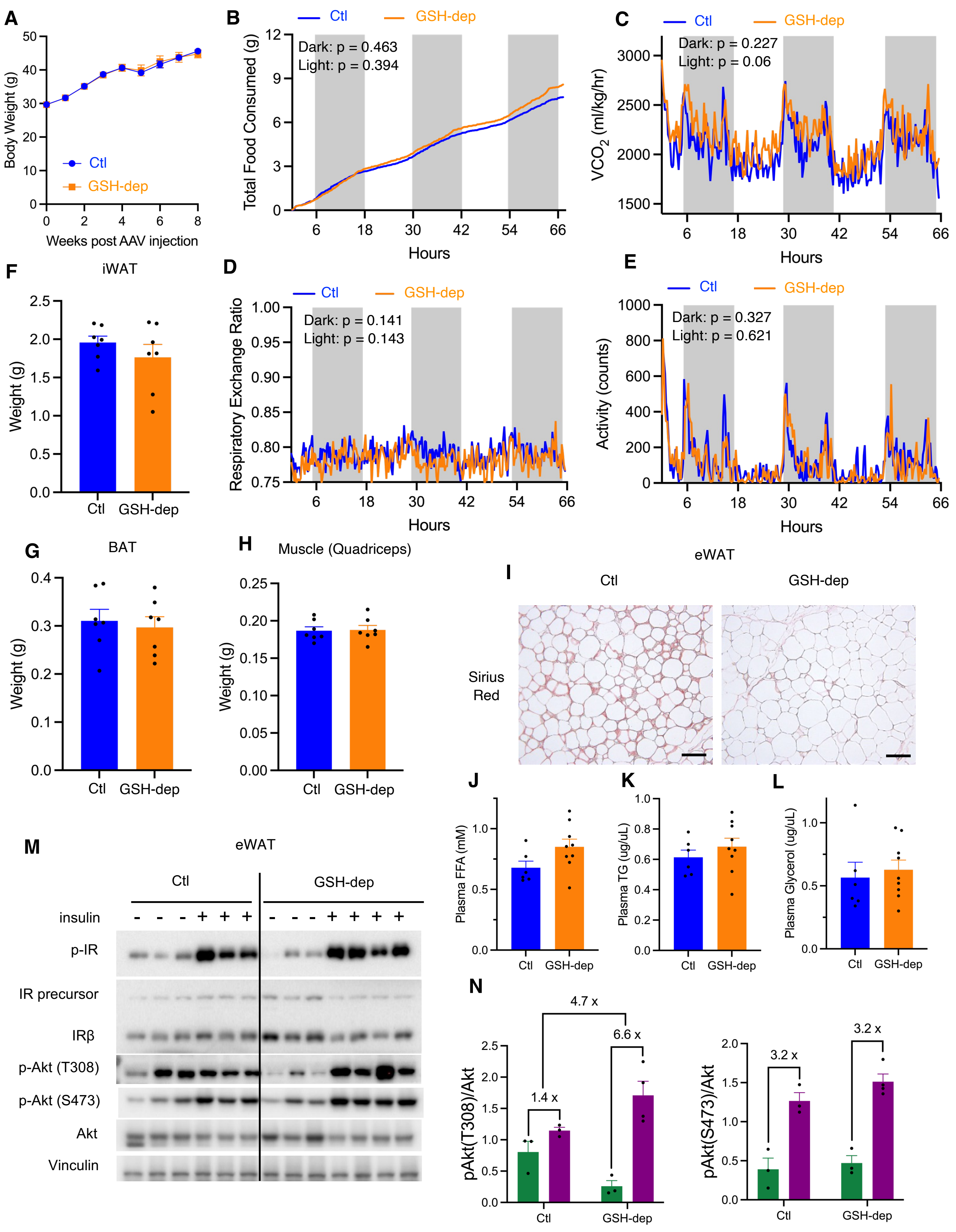

A
Body Weight (g)
Weeks post AAV injection
Ctl
GSH-dep
B
Total Food Consumed (g)
Dark: p = 0.463
Light: p = 0.394
Hours
C
VCO2 (ml/kg/hr)
Dark: p = 0.227
Light: p = 0.06
Hours
D
Respiratory Exchange Ratio
Dark: p = 0.141
Light: p = 0.143
Hours
E
Activity (counts)
Dark: p = 0.327
Light: p = 0.621
Hours
F
iWAT
Weight (g)
G
BAT
Weight (g)
H
Muscle (Quadriceps)
Weight (g)
I
eWAT
Ctl
GSH-dep
Sirius Red
J
Plasma FFA (mM)
K
Plasma TG (ug/uL)
L
Plasma Glycerol (ug/uL)
M
eWAT
Ctl
GSH-dep
insulin
p-IR
IR precursor
IRβ
p-Akt (T308)
p-Akt (S473)
Akt
Vinculin
N
pAkt(T308)/Akt
4.7 x
6.6 x
1.4 x
pAkt(S473)/Akt
3.2 x
3.2 x

Figure S2. Hepatic GSH depletion protects against MASLD.
(A) Body weights of mice post AAV injection. (n = 7-8)
(B) Cumulative food intake by control or GSH depleted mice. (n = 5)
(C) Rate of carbon dioxide production of mice. (n = 5)
(D) Respiratory exchange ratio of mice measured by metabolic cage. (n = 5)
(E) Activities of mice across the circadian cycle. (n = 5)
(F-H) Tissue weights in control or GSH depleted mice under HFD.
(I) Sirius Red staining showing the fibrosis in eWAT of control or GSH depleted mice under HFD. Scale bar: 80 μm.
(J-L) Circulating levels of free fatty acid (FFA) (J), triglyceride (TG) (K), and glycerol (L) in mice.
(M) Protein levels of key downstream effectors of insulin signaling in eWAT after insulin injection through vena cava.
(N) Quantitative analysis of immunoblots in (M).
GSH-dep: GSH depletion. Mice were fed a high-fat diet for 2 weeks before infection with AAV, and were sacrificed 8 weeks later.

Figure S3

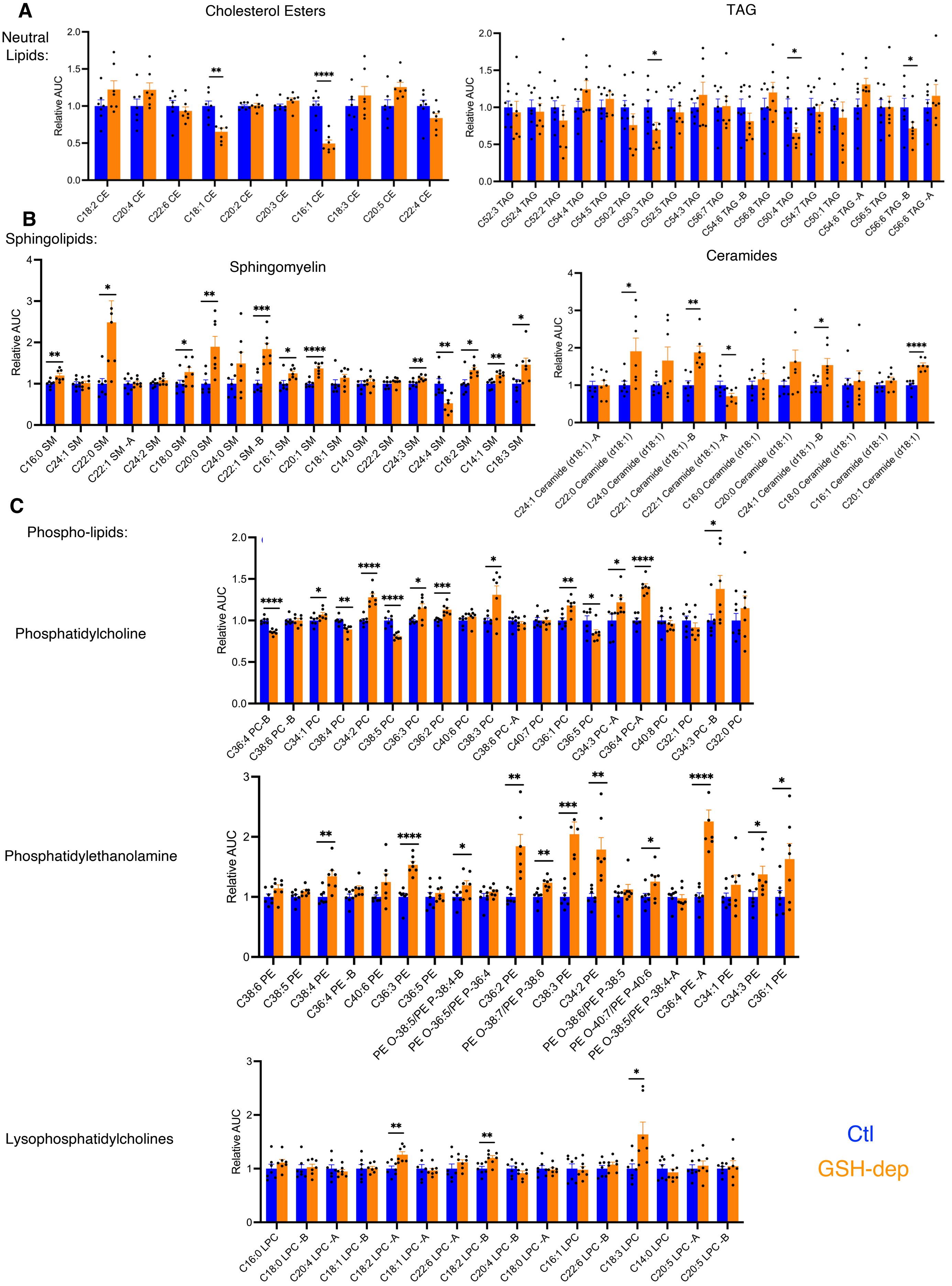
A
Neutral Lipids:
Cholesterol Esters
TAG
Relative AUC
C18:2 CE
C20:4 CE
C22:6 CE
C18:1 CE
C20:2 CE
C20:3 CE
C16:1 CE
C18:3 CE
C20:5 CE
C22:4 CE
C52:3 TAG
C52:4 TAG
C52:2 TAG
C54:4 TAG
C54:5 TAG
C50:2 TAG
C50:3 TAG
C52:5 TAG
C54:3 TAG
C56:7 TAG
C54:6 TAG -B
C56:8 TAG
C50:4 TAG
C54:7 TAG
C50:1 TAG
C54:6 TAG -A
C56:5 TAG
C56:6 TAG -B
C56:6 TAG -A
B
Sphingolipids:
Sphingomyelin
Ceramides
C16:0 SM
C24:1 SM
C22:0 SM
C22:1 SM -A
C24:2 SM
C18:0 SM
C20:0 SM
C24:0 SM
C22:1 SM -B
C16:1 SM
C20:1 SM
C18:1 SM
C14:0 SM
C22:2 SM
C24:3 SM
C24:4 SM
C18:2 SM
C14:1 SM
C18:3 SM
C24:1 Ceramide (d18:1) -A
C22:0 Ceramide (d18:1)
C24:0 Ceramide (d18:1)
C22:1 Ceramide (d18:1) -B
C22:1 Ceramide (d18:1) -A
C16:0 Ceramide (d18:1)
C20:0 Ceramide (d18:1)
C24:1 Ceramide (d18:1) -B
C18:0 Ceramide (d18:1)
C16:1 Ceramide (d18:1)
C20:1 Ceramide (d18:1)
C
Phospho-lipids:
Phosphatidylcholine
C36:4 PC-B
C38:6 PC -B
C34:1 PC
C38:4 PC
C34:2 PC
C38:5 PC
C36:3 PC
C36:2 PC
C40:6 PC
C38:3 PC
C38:6 PC -A
C40:7 PC
C36:1 PC
C36:5 PC
C34:3 PC -A
C36:4 PC-A
C40:8 PC
C32:1 PC
C34:3 PC -B
C32:0 PC
Phosphatidylethanolamine
C38:6 PE
C38:5 PE
C38:4 PE
C36:4 PE -B
C40:6 PE
C36:3 PE
C36:5 PE
PE O-38:5/PE P-38:4-B
PE O-36:5/PE P-36:4
C36:2 PE
PE O-38:7/PE P-38:6
C38:3 PE
C34:2 PE
PE O-38:6/PE P-38:5
PE O-40:7/PE P-40:6
PE O-38:5/PE P-38:4-A
C36:4 PE -A
C34:1 PE
C34:3 PE
C36:1 PE
Lysophosphatidylcholines
C16:0 LPC
C18:0 LPC -B
C20:4 LPC -A
C18:1 LPC -B
C18:2 LPC -A
C18:1 LPC -A
C22:6 LPC -A
C18:2 LPC -B
C20:4 LPC -B
C18:0 LPC -A
C16:1 LPC
C22:6 LPC -B
C18:3 LPC
C14:0 LPC
C20:5 LPC -A
C20:5 LPC -B
Ctl
GSH-dep

Figure S3. Hepatic lipid profile of control and GSH depleted mice.
(A) Levels of cholesterol esters and triacylglycerols in mouse livers under HFD. Lipids with -A and -B means detected isomers with two peaks.
(B) Levels of sphingomyelin and ceramides in mouse livers under HFD. Lipids with -A and -B means detected isomers with two peaks.
(C) Levels of phosphatidylcholine, phosphatidylethanolamine, and lysophosphatidylcholines in mouse livers under HFD. Lipids with -A and -B means detected isomers with two peaks.
GSH-dep: GSH depletion. Mice were fed a high-fat diet for 2 weeks before infection with AAV, and were sacrificed 8 weeks later.

Figure S4

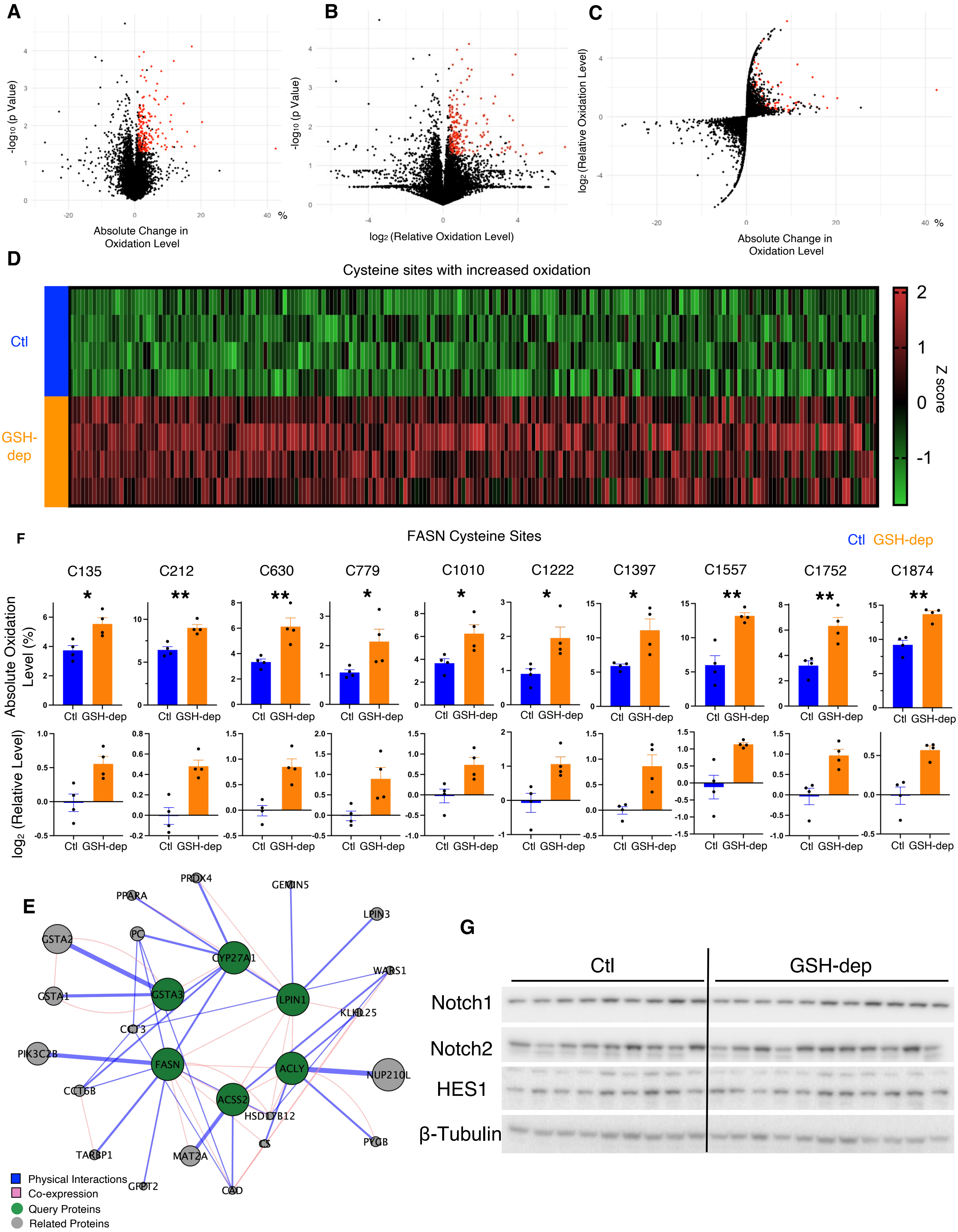
A
-log10 (p Value)
Absolute Change in Oxidation Level
%
B
-log10 (p Value)
log2 (Relative Oxidation Level)
C
log2 (Relative Oxidation Level)
Absolute Change in Oxidation Level
%
D
Cysteine sites with increased oxidation
Ctl
GSH-dep
Z score
F
FASN Cysteine Sites
Ctl GSH-dep
C135
C212
C630
C779
C1010
C1222
C1397
C1557
C1752
C1874
Absolute Oxidation Level (%)
log2 (Relative Level)
Ctl GSH-dep
E
PRDX4
GEMIN5
PPARA
LPIN3
GSTA2
PC
CYP27A1
WARS1
GSTA1
GSTA3
LPIN1
KLHL25
CCT3
PIK3C2B
FASN
ACLY
NUP210L
CCT6B
ACSS2
HSD17B12
TARBP1
MAT2A
CS
PYGB
GPT2
CAD
Physical Interactions
Co-expression
Query Proteins
Related Proteins
G
Ctl
GSH-dep
Notch1
Notch2
HES1
β-Tubulin

Figure S4. Differentially oxidized cysteine sites induced by GSH depletion.
(A) Scatter plot showing the absolute changes and p values for oxidation levels of detected cysteine sites following GSH depletion. Red: cysteine sites with increased level of oxidation.
(B) Scatter plot showing the log relative and p values for oxidation levels of detected cysteine sites following GSH depletion. Red: cysteine sites with increased level of oxidation.
(C) Scatter plot showing the log and absolute changes for oxidation levels of detected cysteine sites following GSH depletion. Red: cysteine sites with increased level of oxidation.
For (A-C), cysteine sites with significantly increased oxidation were defined as those with a p-value < 0.05, a relative oxidation increase of at least 25%, and falling within the top 15% of all sites ranked by the absolute increase of oxidation level.
(D) Heatmap showing z scores of oxidation levels of cysteine sites with increased levels of oxidation following GSH depletion.
(E) Interactome of proteins in lipid metabolic process containing cysteine sites with increased oxidation following GSH depletion. The analysis was performed using GeneMANIA.
(F) Oxidation levels of cysteine sites with increased level of oxidation in fatty acid synthase (FASN).
(G) Protein levels of key effectors of Notch signaling in liver. The β-Tubulin loading control shown is identical to that in Fig. 1C, as the same protein lysates were analyzed.
GSH-dep: GSH depletion.

Figure S5

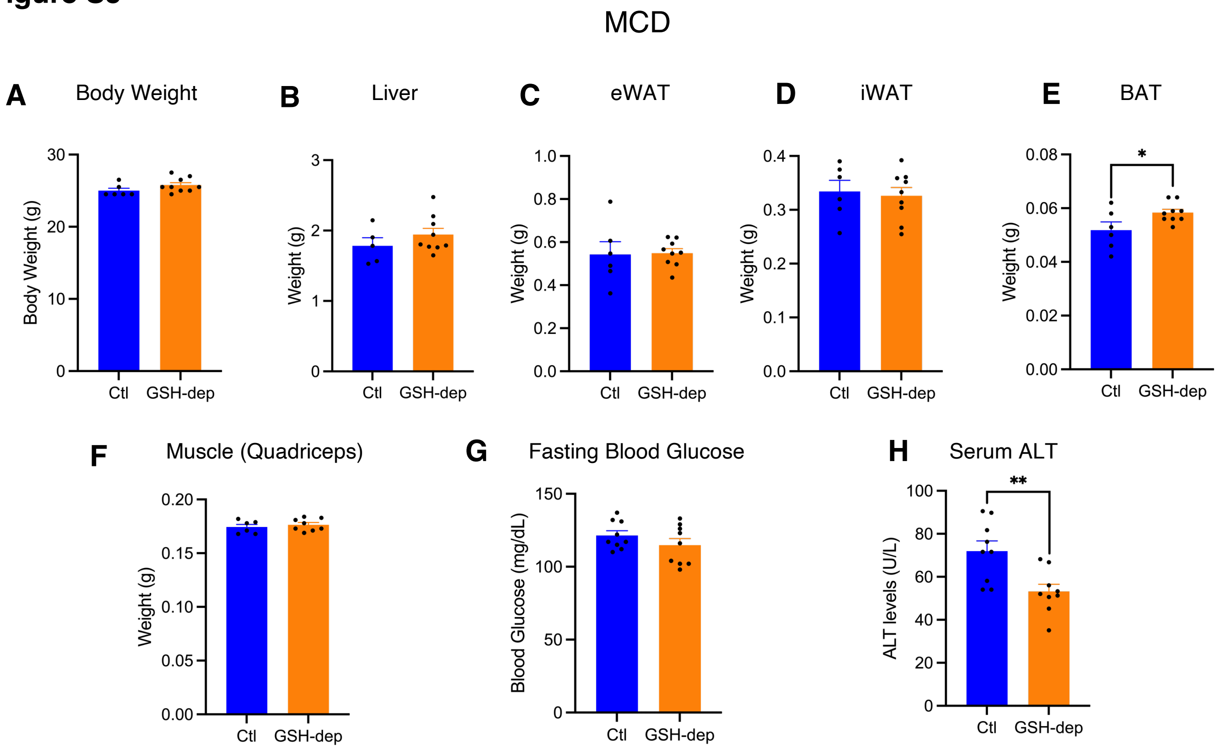

Figure S5. Hepatic GSH depletion under Methionine-Choline Deficient (MCD) Diet.
(A) Body weights of mice 8 weeks after AAV injection.
(B-F) Tissue weights of mice 8 weeks after AAV injection.
(G) Blood glucose levels of mice after 3-hour fasting.
(H) Circulating levels of ALT in mice.
GSH-dep: GSH depletion. Mice were fed an MCD diet after infection with AAV, and were sacrificed 8 weeks later.

Figure S6

A

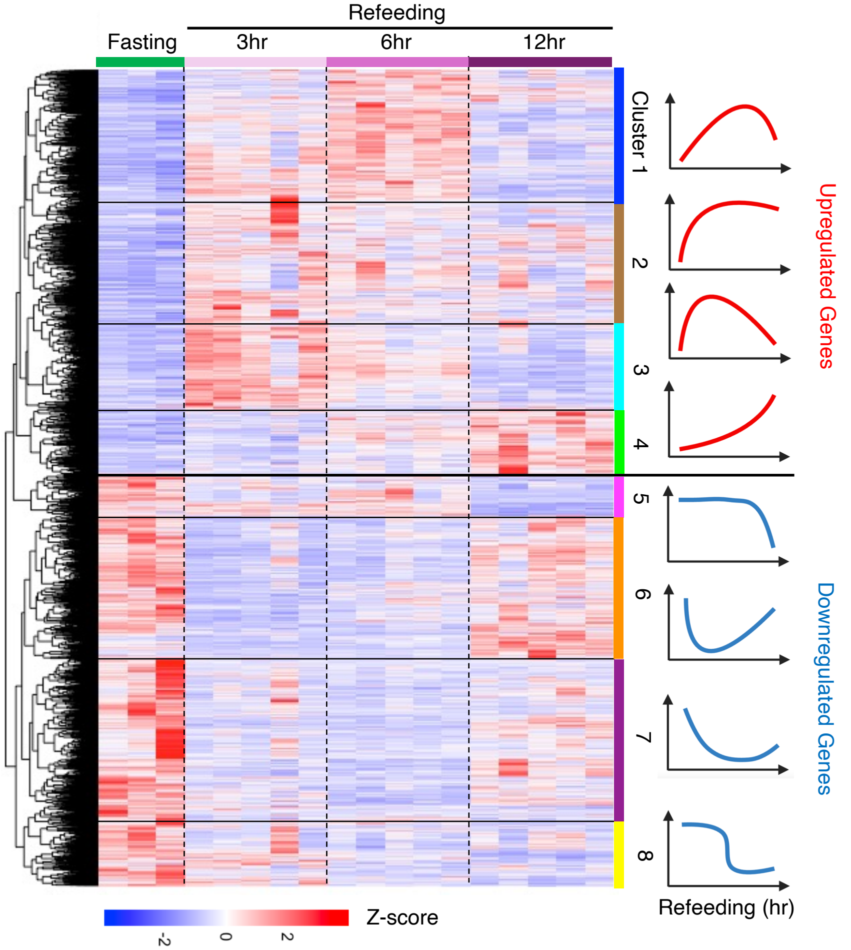

B

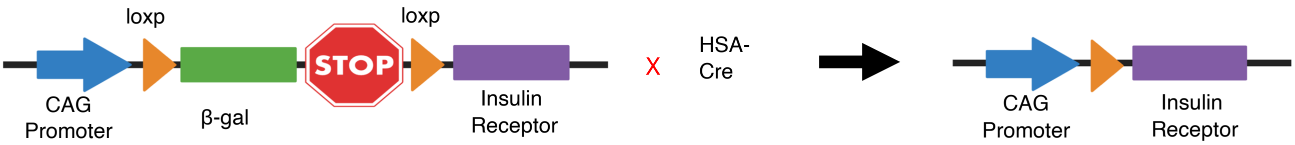

C

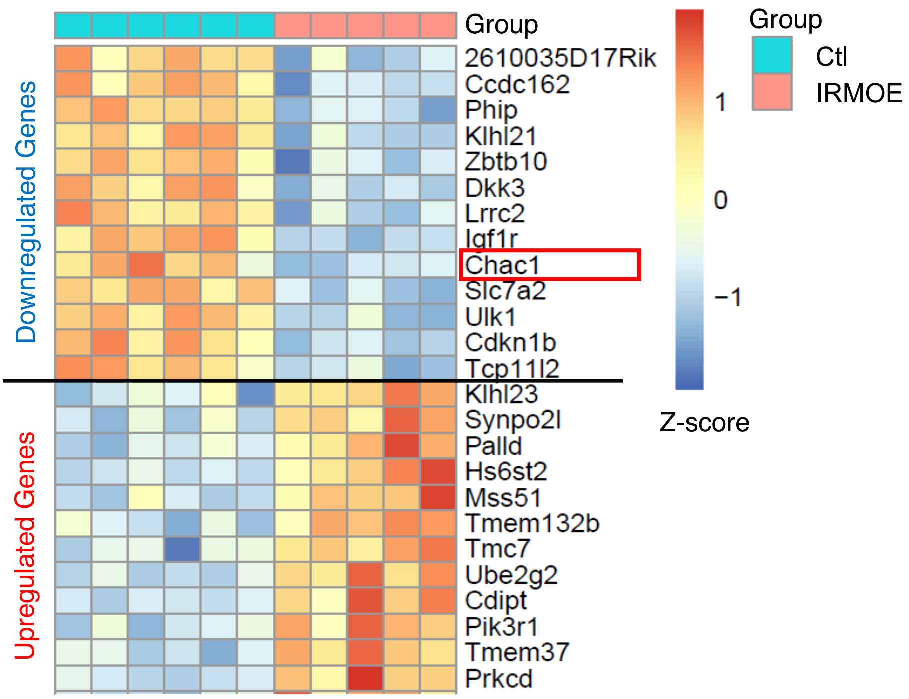

Figure S6. Chac1 is regulated during feeding and changes in insulin sensitivity.
(A) Cluster analysis of differentially expressed genes in mouse livers under fasting or following refeeding. Raw data were from GSE137385. (n= 3-5)
(B) Schematic showing the construction of mice with muscle-specific insulin receptor overexpression (IRMOE). CAG: cytomegalovirus (CMV) enhancer fused to the chicken beta-actin promoter; HAS: human α-skeletal actin promoter.
(C) The top 25 IRMOE-regulated genes in skeleton muscle, ranked by p value.

Figure S7

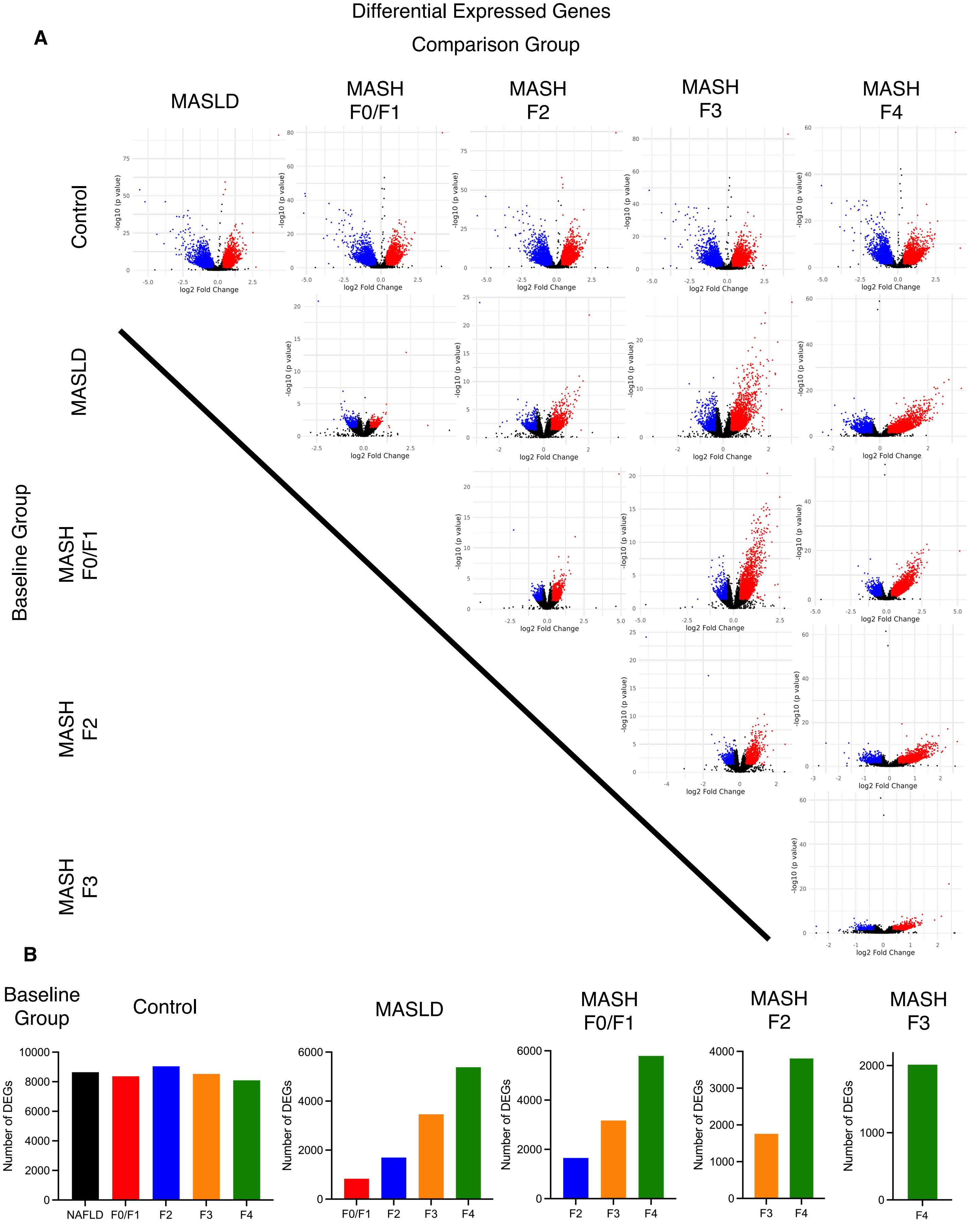
Differential Expressed Genes
A
Comparison Group
MASLD
MASH F0/F1
MASH F2
MASH F3
MASH F4
Baseline Group
Control
MASLD
MASH F0/F1
MASH F2
MASH F3
-log10 (p value)
log2 Fold Change
B
Baseline Group
Control
MASLD
MASH F0/F1
MASH F2
MASH F3
Number of DEGs
NAFLD
F0/F1
F2
F3
F4

Figure S7. Trajectory of transcriptional reprogramming during the development of MASLD/MASH.

(A) Volcano plots showing differential gene expression between two groups during the development of MASLD/MASH.

(B) Numbers of differentially expressed genes between two groups during the development of MASLD/MASH.

Figure S8

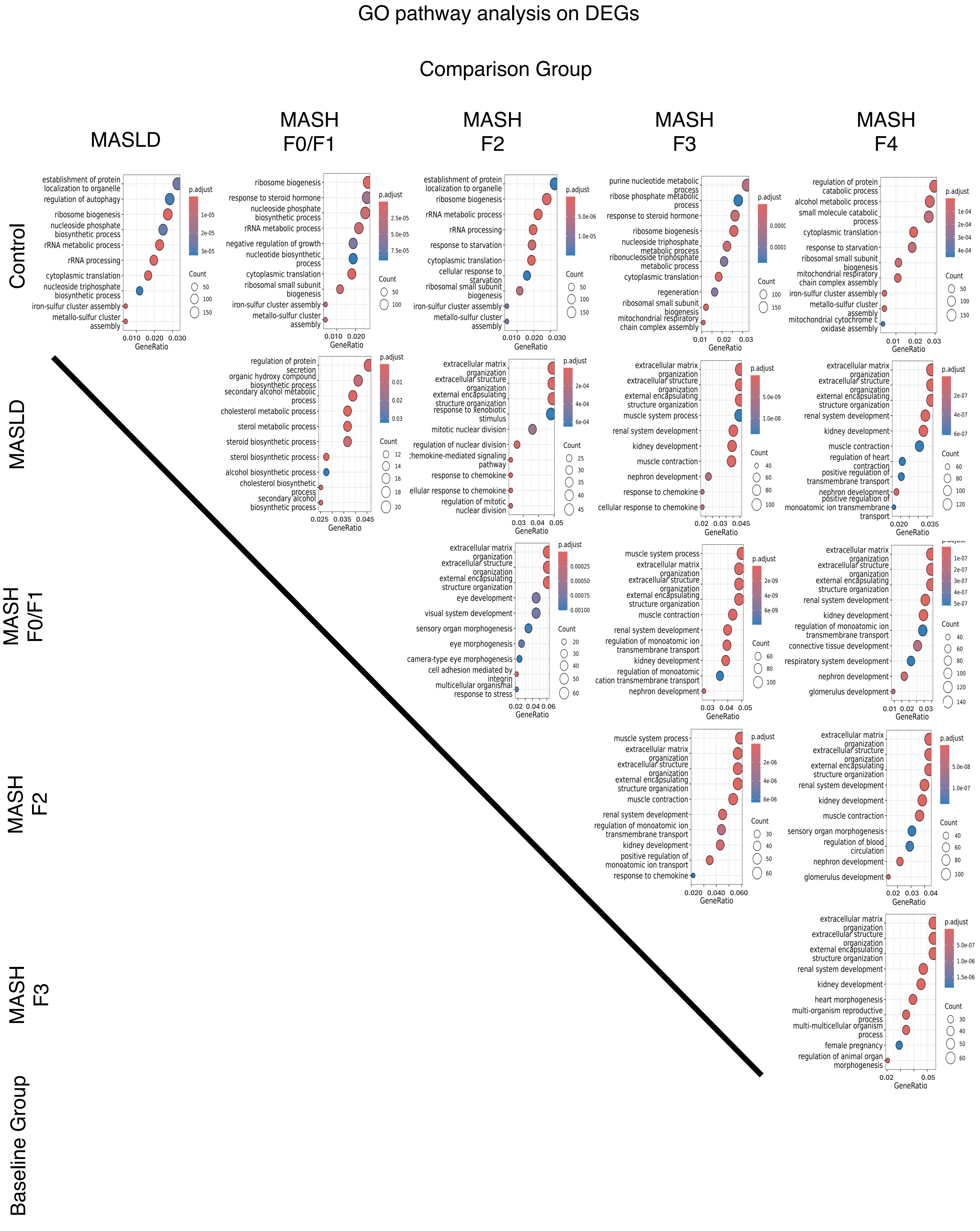

GO pathway analysis on DEGs
Comparison Group
MASLD
MASH F0/F1
MASH F2
MASH F3
MASH F4
Control
MASLD
MASH F0/F1
MASH F2
MASH F3
Baseline Group
GeneRatio
p.adjust
Count

Figure S8. Pathways involved in the development of MASLD/MASH.
Gene ontology (GO) pathway analysis of differentially expressed genes between two groups during the development of MASLD/MASH.
