# Supplementary material for: Hepatic glutathione depletion ameliorates MASLD through selective protein oxidation and inhibition of lipogenesis
Source: J Clin Invest. 2026 Apr 15;136(8):e197556. doi: 10.1172/JCI197556 (PMC13078873; doi:10.1172/JCI197556)

1c

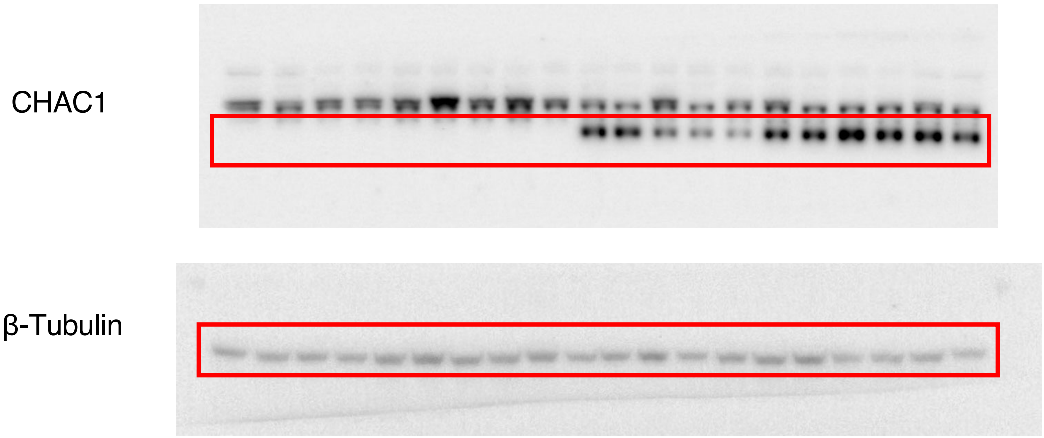
CHAC1
β-Tubulin

2m

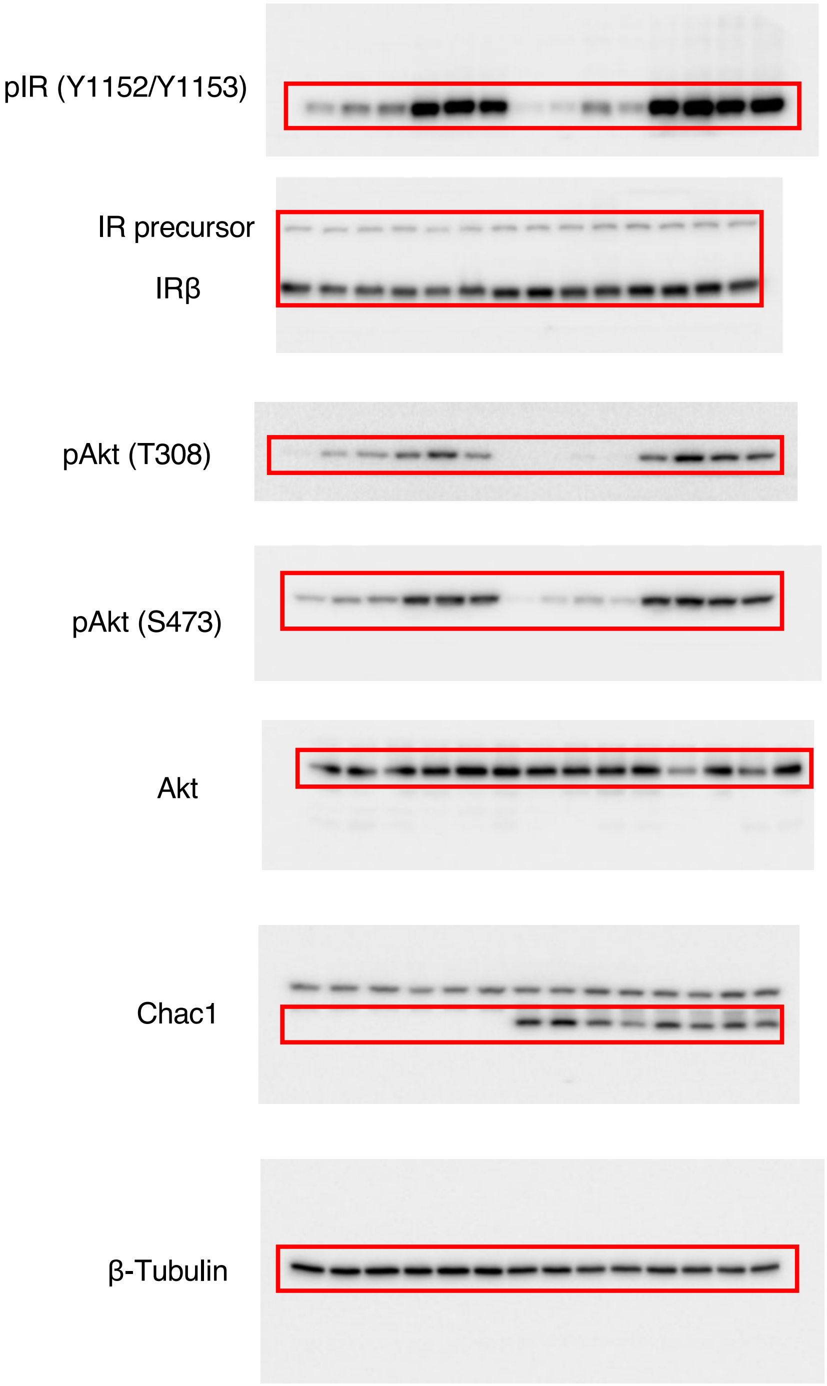
pIR (Y1152/Y1153)
IR precursor
IRβ
pAkt (T308)
pAkt (S473)
Akt
Chac1
β-Tubulin

2o

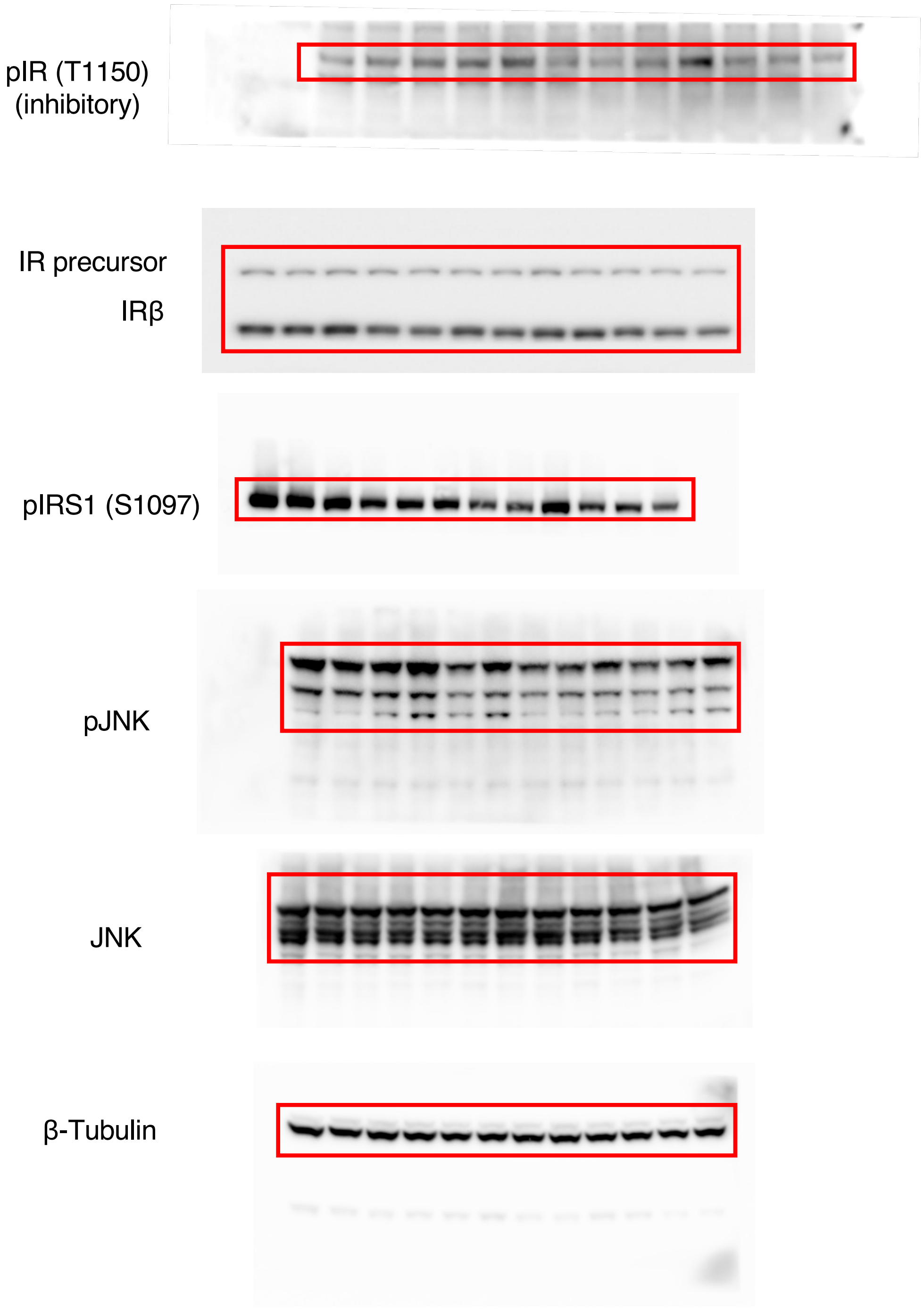
pIR (T1150)
(inhibitory)
IR precursor
IRβ
pIRS1 (S1097)
pJNK
JNK
β-Tubulin

3c

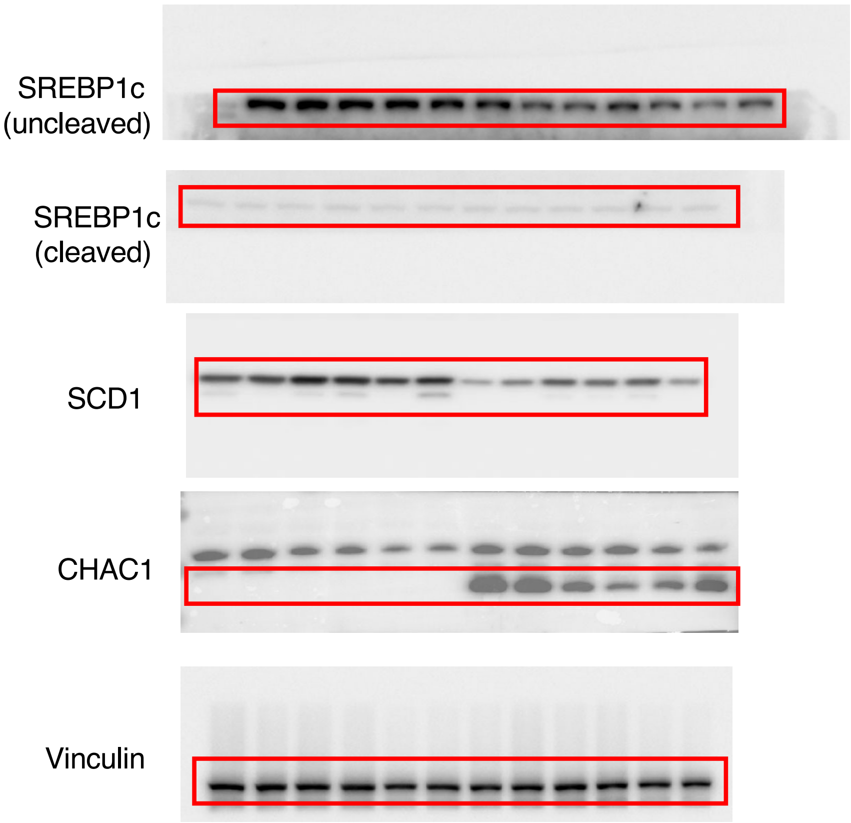

SREBP1c
(uncleaved)
SREBP1c
(cleaved)
SCD1
CHAC1
Vinculin

3e

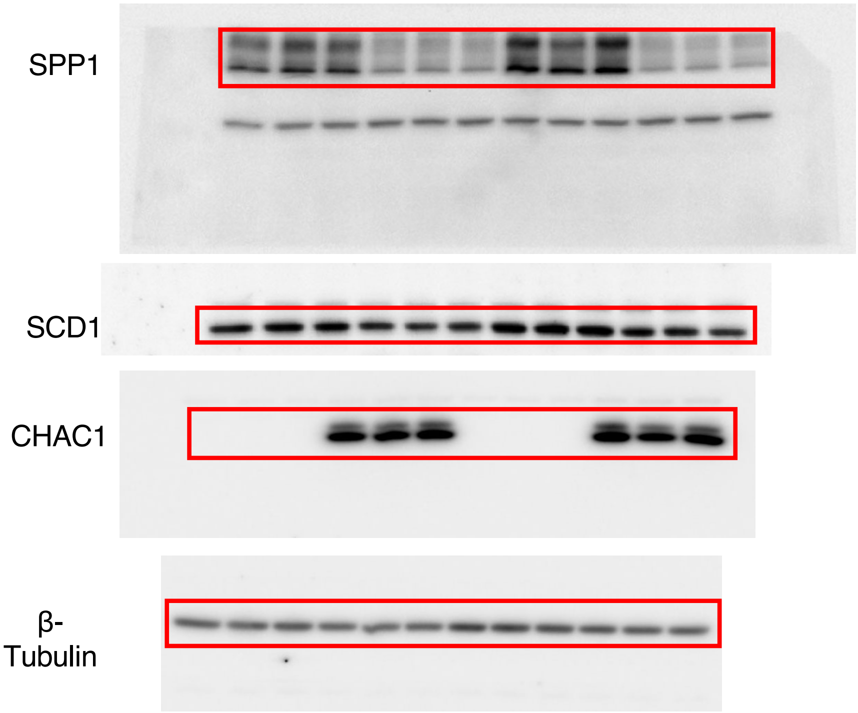

SPP1
SCD1
CHAC1
β-
Tubulin

4h

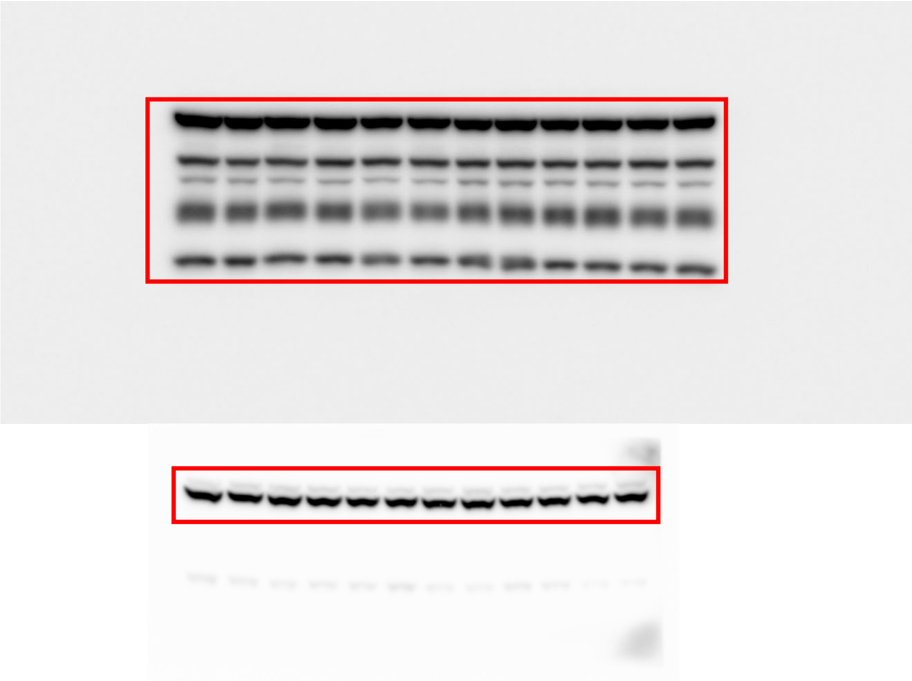

OXPHOS

ATP5A

UQCRC2

MTCO1

SDHB

β-tubulin
(same as
2o)

5i

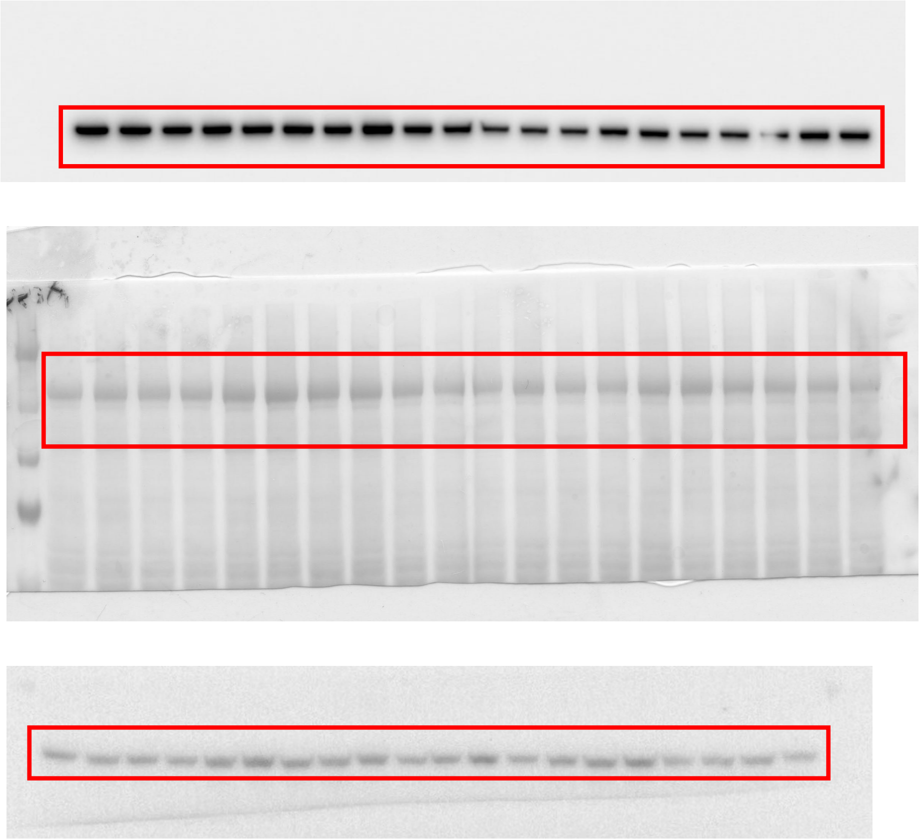

FASN

Ponceau
S

β-Tubulin
(same as 1c)

5I

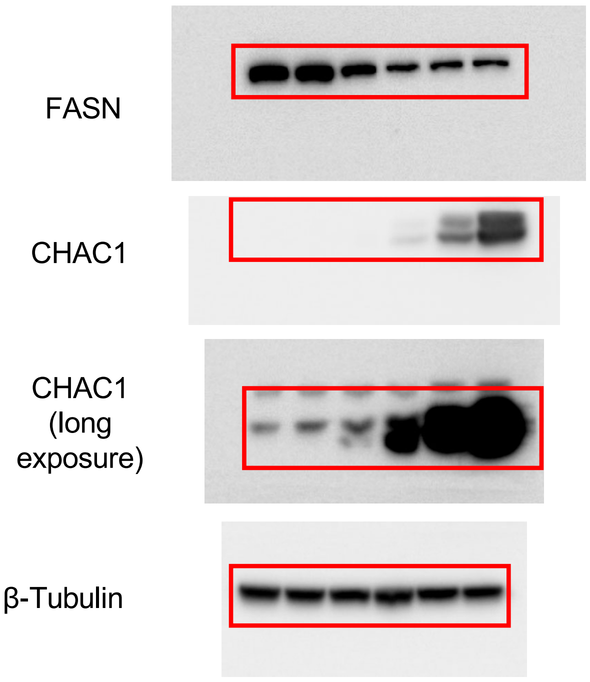
FASN
CHAC1
CHAC1 (long exposure)
β-Tubulin

S2m

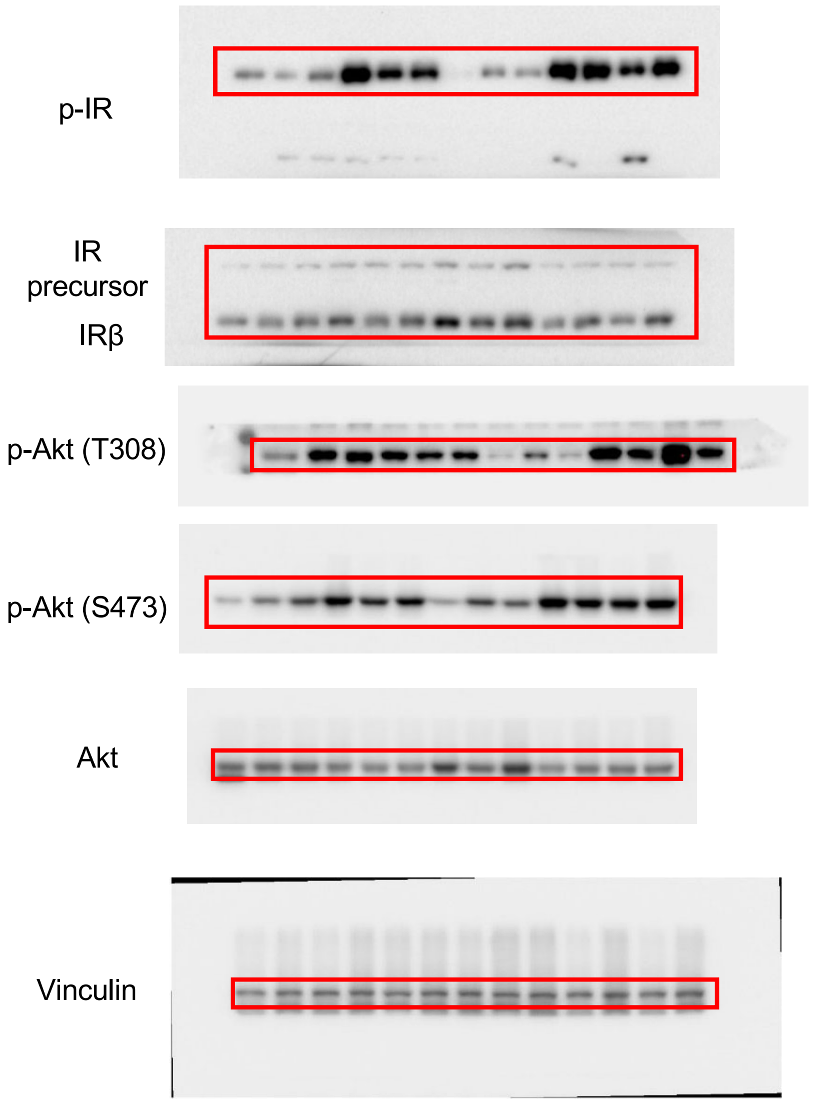
p-IR
IR precursor
IRβ
p-Akt (T308)
p-Akt (S473)
Akt
Vinculin

S4g

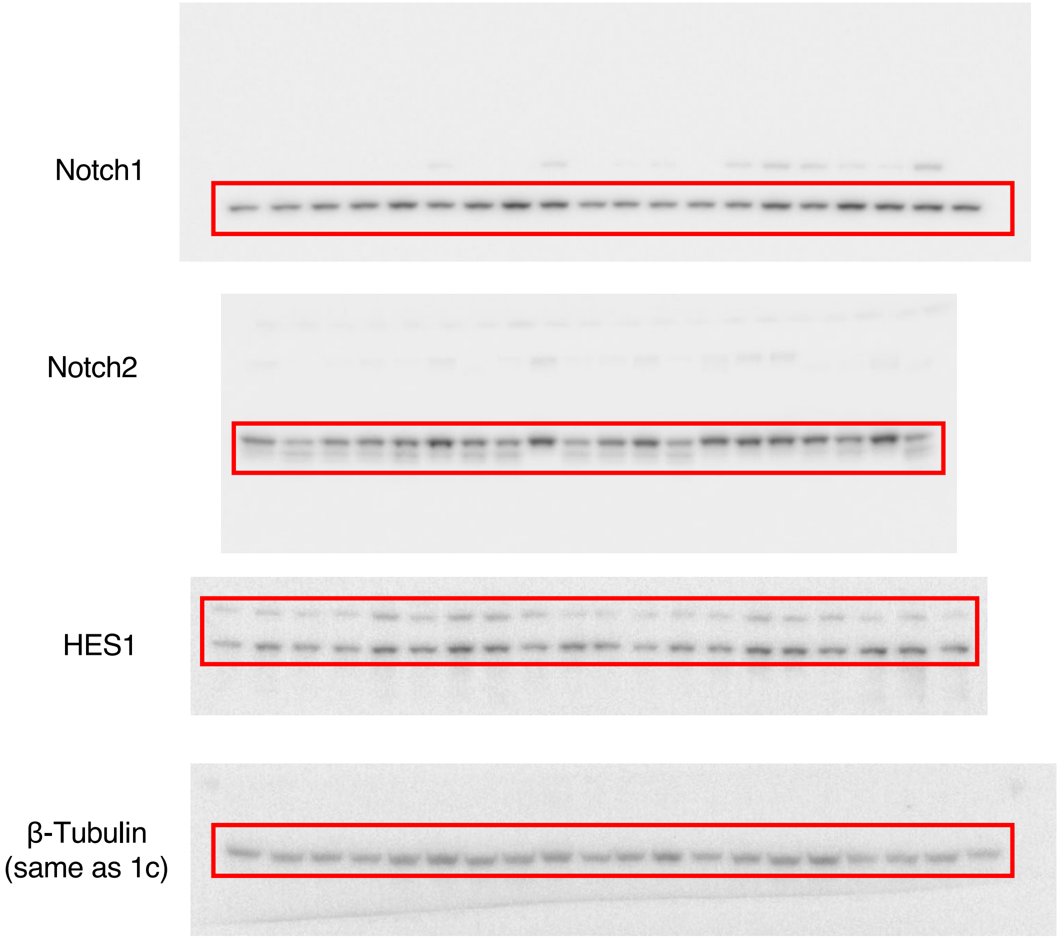

Supplement: Unedited blot and gel images [file jci-136-197556-s117.pdf]
